# Tryptophan metabolism is inversely regulated in the tumor and blood of patients with glioblastoma

**DOI:** 10.7150/thno.60679

**Published:** 2021-09-03

**Authors:** Verena Panitz, Saša Končarević, Ahmed Sadik, Dennis Friedel, Tobias Bausbacher, Saskia Trump, Vadim Farztdinov, Sandra Schulz, Philipp Sievers, Stefan Schmidt, Ina Jürgenson, Stephan Jung, Karsten Kuhn, Irada Pflüger, Suraj Sharma, Antje Wick, Pauline Pfänder, Stefan Selzer, Philipp Vollmuth, Felix Sahm, Andreas von Deimling, Ines Heiland, Carsten Hopf, Peter Schulz-Knappe, Ian Pike, Michael Platten, Wolfgang Wick, Christiane A. Opitz

**Affiliations:** 1 DKTK Brain Cancer Metabolism Group, German Cancer Research Center (DKFZ), 69120 Heidelberg, Germany.; 2 Department of Neurology and National Center for Tumor Diseases, Heidelberg University Hospital, 69120 Heidelberg, Germany.; 3 Proteome Sciences R&D GmbH & Co. KG, Altenhöferallee 3, 60438 Frankfurt/Main, Germany.; 4 Faculty of Bioscience, Heidelberg University, 69120 Heidelberg, Germany.; 5 Center for Mass Spectrometry and Optical Spectroscopy (CeMOS), Mannheim University of Applied Sciences, 68163 Mannheim, Germany.; 6 Molecular Epidemiology Unit, Berlin Institute of Health at Charité - Universitätsmedizin Berlin, 10117 Berlin, Germany.; 7 Department of Neuropathology, Institute of Pathology, Heidelberg University Hospital, 69120 Heidelberg, Germany.; 8 Clinical Cooperation Unit Neuropathology, German Consortium for Translational Cancer Research (DKTK), German Cancer Research Center (DKFZ), 69120 Heidelberg, Germany.; 9 Department of Neuroradiology, Heidelberg University Hospital, 69120 Heidelberg, Germany.; 10 Department of Arctic and Marine Biology, UiT, The Arctic University of Norway, 9037 Tromsø, Norway.; 11 Proteome Sciences plc, 5 Dashwood Lang Road, Bourne Business Park, Addlestone, Surrey KT15 2HJ, United Kingdom.; 12 Department of Neurology, Medical Faculty Mannheim, University of Heidelberg, 68167 Mannheim, Germany.; 13 DKTK Clinical Cooperation Unit Neuroimmunology and Brain Tumor Immunology, German Cancer Research Center (DKFZ), 69120 Heidelberg, Germany.; 14 Clinical Cooperation Unit Neurooncology, German Cancer Research Center (DKFZ), 69120 Heidelberg, Germany.

**Keywords:** Glioblastoma, tryptophan, AHR, mass spectrometry, MALDI MSI

## Abstract

Tryptophan (Trp)-catabolic enzymes (TCEs) produce metabolites that activate the aryl hydrocarbon receptor (AHR) and promote tumor progression and immunosuppression in glioblastoma. As therapies targeting TCEs or AHR become available, a better understanding of Trp metabolism is required.

**Methods:** The combination of LC-MS/MS with chemical isobaric labeling enabled the simultaneous quantitative comparison of Trp and its amino group-bearing metabolites in multiple samples. We applied this method to the sera of a cohort of 43 recurrent glioblastoma patients and 43 age- and sex-matched healthy controls. Tumor volumes were measured in MRI data using an artificial neural network-based approach. MALDI MSI visualized Trp and its direct metabolite *N*-formylkynurenine (FK) in glioblastoma tissue. Analysis of scRNA-seq data was used to detect the presence of Trp metabolism and AHR activity in different cell types in glioblastoma.

**Results:** Compared to healthy controls, glioblastoma patients showed decreased serum Trp levels. Surprisingly, the levels of Trp metabolites were also reduced. The decrease became smaller with more enzymatic steps between Trp and its metabolites, suggesting that Trp availability controls the levels of its systemic metabolites. High tumor volume associated with low systemic metabolite levels and low systemic kynurenine levels associated with worse overall survival. MALDI MSI demonstrated heterogeneity of Trp catabolism across glioblastoma tissues. Analysis of scRNA-seq data revealed that genes involved in Trp metabolism were expressed in almost all the cell types in glioblastoma and that most cell types, in particular macrophages and T cells, exhibited AHR activation. Moreover, high AHR activity associated with reduced overall survival in the glioblastoma TCGA dataset.

**Conclusion:** The novel techniques we developed could support the identification of patients that may benefit from therapies targeting TCEs or AHR activation.

## Introduction

Glioblastoma is the most common and most aggressive primary brain cancer in adults. Despite standard of care, comprising maximal safe surgical resection and subsequent radio-chemotherapy with the alkylating agent temozolomide, patients with glioblastoma have a dismal prognosis with a median overall survival of only 15 months and less than 5% of patients being alive five years after primary diagnosis 1-3. Recent controlled clinical trials exploring targeted and immune therapies have not been demonstrated to improve overall survival 3. The high intertumoral 4 and intratumoral heterogeneity 5, 6, the plasticity of glioblastoma cells 7, 8 and a highly immunosuppressive tumor microenvironment (TME) 9 consisting of macrophages, microglia and MDSCs 10-22 all contribute to the high therapy resistance of this cancer entity.

Tryptophan (Trp) metabolism represents an important mechanism mediating therapy resistance in glioblastoma and other tumor entities 23. The Trp-catabolic enzymes (TCEs), indoleamine-2,3-dioxygenase 1 and 2 (IDO1 and IDO2) and tryptophan-2,3-dioxygenase (TDO2), which mediate the first step of the kynurenine pathway (KP), are upregulated in diverse cancer entities. IDO1 and TDO2 expression have previously been shown in glioblastoma cell lines 24-29, and in glioblastoma tissue 26, 27, 30-33. Two mechanisms by which these TCEs contribute to immunosuppression and tumor progression have been proposed: On the one hand, the degradation of Trp can deplete cells and their environment of this essential amino acid thereby mediating immunosuppressive effects in the TME. On the other hand, Trp degradation produces bioactive downstream metabolites. These metabolites, such as kynurenine (Kyn) and kynurenic acid, can activate the aryl hydrocarbon receptor (AHR), a ligand-activated transcription factor that induces the transcription of AHR target genes 34. In glioblastoma, high TDO2 expression leads to the production of Kyn, which activates the AHR, thereby enhancing cancer cell motility and limiting the proliferation and function of immune cells in the TME 27. New treatment options targeting IDO1, TDO2 and the AHR 35 are currently tested in clinical trials in diverse tumor entities. Clinical trials of IDO1 inhibitors in glioblastoma are in phase I and II 36. A non-randomized phase I clinical trial in 17 recurrent glioma patients with the IDO1 inhibitor PF-06840003 showed no responses but disease stabilization in 47% of the patients 37. In general, TDO2 inhibitors are in preclinical development and combined IDO1/TDO2 inhibitors are in phase I studies. Phase I clinical trials assess AHR inhibitors in advanced solid tumors 35, 38, 39. Of note, also other therapies modulate Trp catabolism: Radiation and chemotherapy can induce IDO1 expression 40, 41 and immune checkpoint blockade (ICB) with nivolumab increases the Kyn/Trp ratio 42 and induces IDO1 43.

Thus, a better understanding of Trp metabolism in glioblastoma is required to address therapy resistance and to select the patients that will benefit from treatments targeting TCEs and AHR.

In this study, we investigated Trp metabolism in glioblastoma. Multiple label-free methods have been employed to measure Trp and its metabolites 44, 45. Here, we introduce a novel multiplex method to measure the levels of Trp and its amino group-bearing downstream metabolites in the sera of recurrent glioblastoma patients and healthy controls. By using amino-reactive isobaric labeling reagents, this novel multiplex method enabled monitoring and comparison of Trp and its metabolites simultaneously in multiple samples in a single liquid chromatography tandem mass spectrometry (LC-MS/MS) run. Trp and surprisingly also its downstream metabolites were decreased in the sera of patients with glioblastoma. In contrast, MALDI mass spectrometry imaging (MSI) demonstrated heterogeneous Trp catabolism in glioblastoma tumor tissues and single cell RNA-sequencing (scRNA-seq) analysis detected Trp metabolism in all cell types present in glioblastoma.

## Materials and Methods

### TCGA and GTEx datasets

TCGAbiolinks 46 was used to download the harmonized HT-Seq counts and fragments per kilobase of transcript per million mapped reads (FPKM) and the clinical information of The Cancer Genome Atlas (TCGA) glioblastoma (GBM) datasets from Genomic Data Commons (GDC) (https://gdc.cancer.gov/). Patient samples characterized as “primary tumor” were retained (*n* = 156). Additional assignment of TCGA GBM patients to their transcriptional subtypes was retrieved from Wang et al. 8. The FPKM values were converted to transcripts per million (TPMs) 47. TPM data of normal brain tissues (*n* = 1671) were downloaded from the Genotype-Tissue Expression (GTEx) dataset (https://gtexportal.org/home/). All TPM values were log_2_ transformed. RNA-seq counts were saved in DGELists 48, and then the expression matrices were filtered to retain genes that had at least 10 counts across all samples. The filtered expression matrices were normalized by trimmed mean of M values (TMM) normalization 49, followed by variance modeling using voom 50.

The correlation between the expression of selected AHR target genes and that of *IDO1* or *TDO2,* using the normalized count matrix of the TCGA GBM dataset, was determined by calculating Pearson's correlation coefficient.

### scRNA-seq data

The smart-seq2 glioblastoma dataset GSE131928 was directly downloaded from the Gene Expression Omnibus (GEO), and the respective metadata was obtained from Neftel et al. 7. All pediatric samples were filtered out resulting in a total number of 20 adult patients and a total of 5742 cells. The TPM expression matrix and the metadata were saved into a SingleCellExperiment object 51. The expression matrix and metadata matrix were further analyzed using *scanpy* 52. In brief, the expression matrix was log transformed (log(TPM+1)), and the top 5000 highly variable genes (HVGs) were selected as described by Satija et al. 53. Principle component analysis was performed on the HVGs and the first six principle components were used to create the embedded neighborhood graph. Clustering of the neighborhood graph was performed by the Louvain algorithm 54, 55, followed by partition-based graph abstraction (PAGA) 56. PAGA generates a simpler representation of the manifold data that remains faithful to the data topology, which is used for the uniform manifold approximation and projection (UMAP) representation 57, 58. Marker genes described for oligodendrocytes, macrophages and T cells were used to define the malignant and different non-malignant cell populations 7.

### Biological process activity scores

The Kyoto Encyclopedia of Genes and Genomes (KEGG) 59 gene set of the tryptophan metabolic pathway (hsa00380) was downloaded from the MSigDb database (v6) 60, in addition to the gene sets describing the six different cell states described in Neftel et al. 7, and the AHR gene signature from Sadik et al. 43. The gene sets were used to estimate a normalized enrichment score for their respective biological process activity (BPA) score 61, 62 in the bulk or scRNA data. For comparing the state of activity of Trp metabolism or AHR activity, the BPA scores were grouped and compared across the TCGA GBM-defined clusters (classical, mesenchymal, and proneural) 8. For the Louvain-defined cell populations the median BPA score was estimated and used for the comparison of Trp metabolism, the different cell states and AHR activity. BPA scores were generated using the macrophage signatures described in Newman et al. 63 to characterize the macrophage populations of the scRNA dataset.

### Software and statistics

The association between AHR activity and patient survival was analyzed by applying a univariate Cox regression model and a multivariate Cox regression model of AHR activity and patient age at diagnosis. For bioinformatics analysis of TCGA, GTEx and scRNA-seq data, unless stated otherwise, all pairwise comparisons were performed using Kruskal-Wallis and Wilcoxon rank-sum tests. All analyses were run in R, version 3.6.1 (https://cran.r-project.org/), and Bioconductor version 3.9 (https://bioconductor.org/). All graphical representations were generated using *ggplot2*, *ggpubr, corrplot, gplots, gridExtra,* and* RcolorBrewer*.

### Mathematical modeling of Trp metabolism

To simulate Trp metabolism in healthy brain tissue and glioblastoma tissue, we used the mathematical model of human Trp metabolism previously published by Stavrum et al. 64. RNA-seq expression data generated from the GTEx and TCGA databases were integrated into the mathematical model as described in Schäuble et al. 65. The steady state calculation of metabolite concentrations and reaction fluxes was done using COPASI 4.28 66. Samples that had missing expression values for genes represented in the mathematical model of Trp metabolism were removed. An additional outlier correction using robust regression and outlier removal (ROUT) 67 was performed with GraphPad Prism software (Version 8.0, GraphPad Software, Inc.). The concentration of extracellular Trp was set to 0.01 mM, corresponding to the upper bound of free Trp concentrations measured in blood 68. Graphical and statistical analyses of model calculated metabolite concentrations were performed with GraphPad Prism software (Version 8.0). Data are mean +/- SEM. Data were analyzed with two-tailed unpaired Student's t test.

### Human serum and tissue samples

Blood samples from patients with recurrent glioblastoma or from healthy controls were taken after informed consent and approval of the local regulatory authorities (ethics board approval S-496/2014). Blood was centrifuged at 4 °C for 10 min at 2000 g to obtain serum. Collected serum was directly transferred on dry ice and was stored at -80 °C until further analysis.

Tissue specimens of patients diagnosed with glioblastoma were obtained from the Institute of Neuropathology, Heidelberg University Hospital, according to the regulations of the Tissue Bank of the National Center for Tumor Diseases (NCT), Heidelberg University Hospital, under the ethics board approvals S-207/2005 and S-322/2019.

### Metabolite measurements with liquid chromatography tandem mass spectrometry (LC-MS/MS) combined with isobaric chemical labeling

#### Tandem mass tag (TMT^®^)-labeling of tryptophan and its metabolites

L-tryptophan (Trp), 5-hydroxy (OH)-L-tryptophan (OH-Trp), kynurenine (Kyn), 3-OH-kynurenine (OH-Kyn), anthranilic acid (AA), and 3-OH-anthranilic acid (OH-AA) (Sigma-Aldrich), and *N*-formylkynurenine (FK) (ChemCruz) were labeled with TMT^®^ reagent 127 (Proteome Sciences) at a concentration of 15 mM in 100 mM triethylammonium bicarbonate (TEAB; Fluka) for 1 h and the labeling reaction was stopped by addition of hydroxylamine (Sigma-Aldrich) to 0.25%. Analytes were diluted as appropriate in 2% acetonitrile (ACN; LichroSolv, VWR), 0.1% formic acid (FA; VWR) and used for mass spectrometry (MS) measurements.

#### Protein precipitation

60 µL of each human serum sample and aliquots of a mixed pooled reference sample made by pooling aliquots of 34 individual samples were precipitated with 9.8 µL of 72% trichloroacetic acid (TCA; Merck) and centrifuged at 14000 rpm at room temperature in the Eppendorf centrifuge 5417R for 10 min. All supernatants were taken off (~50 µL) and neutralized with 15 µL of 1M sodium hydroxide (NaOH; Kraft). Then 1M TEAB (Sigma) was added to a final concentration of 100 mM. Neutralized samples were stored at -80 °C until labeling.

#### TMT^®^-labeling

TMT^®^-labeling was performed in four batches of each six TMT^®^ sixplexes on two days. The mixed reference serum samples were labeled with TMT^®^ 131. TMT^®^ 127 was reserved for spiking TMT^®^-labeled pure substances OH-Trp, OH-Kyn, AA, and OH-AA, and FK to aid assignment of the peak signals where needed. Sera from tumor and control subjects were randomized over the remaining four channels 126, 128, 129, and 130 with two sera from tumor patients and two sera from controls per plex.

The respective TMT^®^ reagent solved in ACN was added to 17.5 mM in the reaction and incubated for 1 h. Aqueous hydroxylamine solution was added to 0.25% and incubated for 30 min. Samples were combined and vials rinsed with 50 µL of 5% ACN, 0.1% trifluoroacetic acid (TFA; Merck) and added to the respective mixtures. The combined volumes were dried in a Speedvac to completion.

#### Purification of the mixed serum samples

Each dried TMT^®^ sixplex analytical sample corresponds to a total equivalent of 300 µL of serum equivalent (5 x 60 µL). The dried samples were solved in 300 µL of water:ACN 95:5 with 0.1% TFA (buffer A) and 150 µL were used for purification by reversed phase chromatography (Nucleosil 120-5 C18 column, 250 mm x 4.6 mm, Macherey-Nagel) using a Waters 2695 HPLC with UV detection at 214 nm. After loading, the sample was washed 4 min with buffer A. Then, the substances were eluted by increasing the ACN content within 40 min to 55%. The flow rate was kept at 1.5 mL/min over the entire gradient. The fractions eluting from 18-30 min were collected, combined, dried and transferred to mass spectrometric analysis.

#### Liquid chromatography mass spectrometry (LC-MS)

Samples were measured on a triple quadrupole system coupled to a nano-LC-II (EASY-nLC II TSQ Vantage system; Thermo Fisher Scientific). The samples were resolved in 150 µL 2% ACN, 0.1% FA (1 µL sample = 1 µL mixed supernatant equivalent).

For MS analysis on the Triple Quadrupole TSQ Vantage system the TMT^®^ sixplex samples were re-suspended in 150 µL 2% ACN/0.1% FA.

For analysis of Trp and Kyn, samples were diluted 1:20 and 2 µL (0.1 µL serum equivalent) were injected into the EASY-nLC II TSQ Vantage system while 10 µL of serum equivalent were used for the other, lower abundant, metabolites.

First, samples were loaded on a 2 cm long (OD 360 µm, ID 100 µm) capillary column filled with 5 µm ReproSil-Pur C18-AQ (Dr. Maisch GmbH) for trapping and clean-up. Then, analytes were separated through a 15 cm long (OD 360 µm, ID 75 µm) capillary column filled with 3 µm ReproSil-Pur C18-AQ (Dr. Maisch GmbH) using a 25 min gradient from 15 to 30% ACN in 0.1% FA at 300 nL/min.

Eluting analytes were ionized by nano electrospray at 1.6 kV. The Triple Quadrupole instrument was operated in positive and SRM mode. Capillary temperature was set to 220 °C. Transition parameters for the respective analytes were as stated in Table S1 to Table S7. Transition scan time was calculated at 24 ms. Peak width (FWHM (full width at half maximum)) for Q1 and Q3 were set to 0.5 and 0.7, respectively. Collision gas pressure was set to 1.8 mTorr, chromfilter peak width to 6.0 s and declustering voltage to 5 V. Mean technical coefficients of variation (CV) for the metabolites measured are shown in Table S8.

#### Processing of SRM data

Data was analyzed in Skyline 3.1 (https://skyline.ms/project/home/software/Skyline/begin.view) 69 using its small molecule capabilities. Fragment ion signals were used for identification verification. Peak integration of TMT^®^ reporter channels was manually edited and areas were exported for further analysis.

#### Statistical analysis of LC-MS generated data

Data integration and statistical analysis was conducted using internally developed software written in R statistical programming language (Version 3.5.2, http://www.R-project.org).

#### Data quality control

The data output of skyline contains the peak area for each analyte and the respective TMT^®^ channel. This “raw” data was used for a bioinformatic quality control. For each analyte the areas of the respective channels of each individual sample or reference were plotted against the median areas of the TMT^®^ sixplexes. In an ideal case the plot is linear and has a linear coefficient of ~1. Low quality reference values were replaced using the median values of channels 126, 128, 129, and 130 for each of the respective analytes.

#### Application of reference design and production of ratio matrix

As to the reference design of the study, ratios of sample/reference were calculated for each analyte of the individual samples. Clinical information of the samples was combined with this analyte matrix.

#### Exploratory analysis

Exploratory analysis was carried out using principal component analysis, heatmaps and unsupervised clustering of samples (http://www.R-project.org, 70, 71). The influence of clinical factors on the data was investigated.

#### Statistical analysis

Prior to statistical analysis 5 pairs of patients and controls had to be excluded due to technical problems or IDH status of patients being mutant. Statistical analysis was conducted using proprietary software FeaST (Proteome Sciences) and was based on linear modeling 72. To evaluate significance of metabolite regulation we applied moderated t-statistics 73, 74 with option for robust empirical Bayes procedure 74. The analysis was carried out using the R package LIMMA 73.

The following linear model was used to identify regulated features among 7 metabolites (Trp, OH-Trp, Kyn, OH-Kyn, AA, OH-AA, FK):

logRatio ~ Class + TMTPlex + channel

Here *logRatio*(i,j) is a matrix of log base 2 ratios log_2_[X(i,j)/X(ref,j)] of sample i feature j abundance to abundance of reference sample feature j. Factor Class describes the influence of disease and has two levels: control and tumor. To account for technical variance (individual samples have been processed and analyzed in different TMT^®^ sixplex experiments) the linear model was extended by inclusion of TMTPlex factor and factor TMT channel. Significance criterion α was set to 0.05.

#### Plotting of individual metabolite values

Graphical analyses of individual sample/reference metabolite values were performed using GraphPad Prism software (Version 8.0). Boxplots represent median, 25^th^ and 75^th^ percentile and whiskers maximum and minimal values.

### MALDI mass spectrometry imaging (MSI)

Glioblastoma tumor tissue samples were cut into 10 μm thick sections, mounted onto ITO slides (Bruker Daltonik) and stored at -80 °C until further processing. For normalization, eleven layers of 5 µM deuterated tryptophan (Trp-D5) dissolved in 50% methanol were deposited with a flow rate of 10 µL/min onto the slides using a SunCollect sprayer (SunChrom). Subsequently, 2,5-dihydroxybenzoic acid (DHB) matrix was prepared at a concentration of 60 mg/mL in ACN/H_2_O/TFA (50:49.5:0.5, v/v/v) and matrix coating was performed with a SunCollect sprayer (SunChrom) in five layers in ascending flow rates (10, 15, 20, 20, 20 μL/min). Data acquisition was performed on a 7T Fourier-transform ion cyclotron resonance (FT-ICR) mass spectrometer (MS) (solariX, Bruker Daltonik). The *m/z* range was set to 150 - 3000 and spectra were acquired at a raster size of 50 µm in positive ion mode by accumulating 200 laser shots per pixel and using a 1 M transient. Spectra were normalized to trp-D5 75 (normalization to peak maximum at *m/z* 210.1285 ± 5 ppm) and visualized using SCiLS Lab Version 2021c Pro (Bruker Daltonik).

### HE-stainings and annotation of tissue regions

Glioblastoma tumor tissue directly adjacent to the tissue mounted for MALDI MSI was cut into 10 μm sections, mounted on Superfrost PLUS slides (Thermo Scientific) and stored at -80 °C until further processing. Hematoxylin-and-eosin (HE)-stainings were performed. Slides were scanned on an Aperio AT2 Scanner (Aperio Technologies) and photographed using Aperio ImageScope software (v12.2.2.5015, Aperio Technologies). Annotation of tissue regions other than tumor tissue, namely highly vascularized tumor tissue or blood, necrosis, infiltration zone, artefacts was performed using QuPath v0.2.3 76 by a clinically experienced neuropathologist.

### Computational analysis of brain tumor volume

The volumetric measurements of MRI data were performed using an artificial neural network (ANN)-based approach, as described previously 77. Briefly, this included brain extraction, followed by image registration and segmentation of contrast-enhanced tumor parts and non-contrast-enhanced T2 weighted/fluid attenuated inversion recovery (FLAIR) signal alterations on MRI. Due to lower data quality like incomplete or corrupt data, 7 of the 43 MRI datasets were analyzed via manual tumor segmentation by a radiologist with 4 years of experience in image processing using ITK-SNAP (www.itksnap.org) 78. Finally, visual inspection was performed for all tumor segmentations to exclude segmentation errors. For each patient, the MRI scan closest to blood sampling was chosen for analysis. Data analysis was approved by the ethics board approvals S-784/2018 and S-496/2014.

### Statistical analyses of clinical characteristics and metabolite levels

To determine whether serum metabolite levels were associated with tumor volumes determined by MRI or the cumulative bevacizumab dose received prior to blood collection regression analyses were applied. Metabolite levels were analyzed separately for their relationship with tumor volume or cumulative bevacizumab dose received before blood draw and considered as dependent variables. Analyses were performed in Statistica version 14.0.0 (Tibco Software Inc.). Based on the standardized b-value and its standard error (SE) derived from Statistica, mean ratios (MR) and 95% confidence intervals (CI) were calculated as follows: MR = e^b^, CI 95% = [e^((b-(2x SE^_b_^)))^, e^((b+(2x SE^_b_^)))^]. Data were plotted using Graph Pad Prism software (Version 8.0).

Two-tailed unpaired Student's t tests were used to analyze potential differences in serum metabolite levels related to molecular features of glioblastoma (MGMT promotor methylation status, EGFR amplification status, PTEN loss) or in association to prior treatment with bevacizumab.

### Data availability

This study includes no data deposited in external repositories.

## Results

### Upregulated Trp metabolism associates with AHR activity in glioblastoma tissue

**Figure 1:**
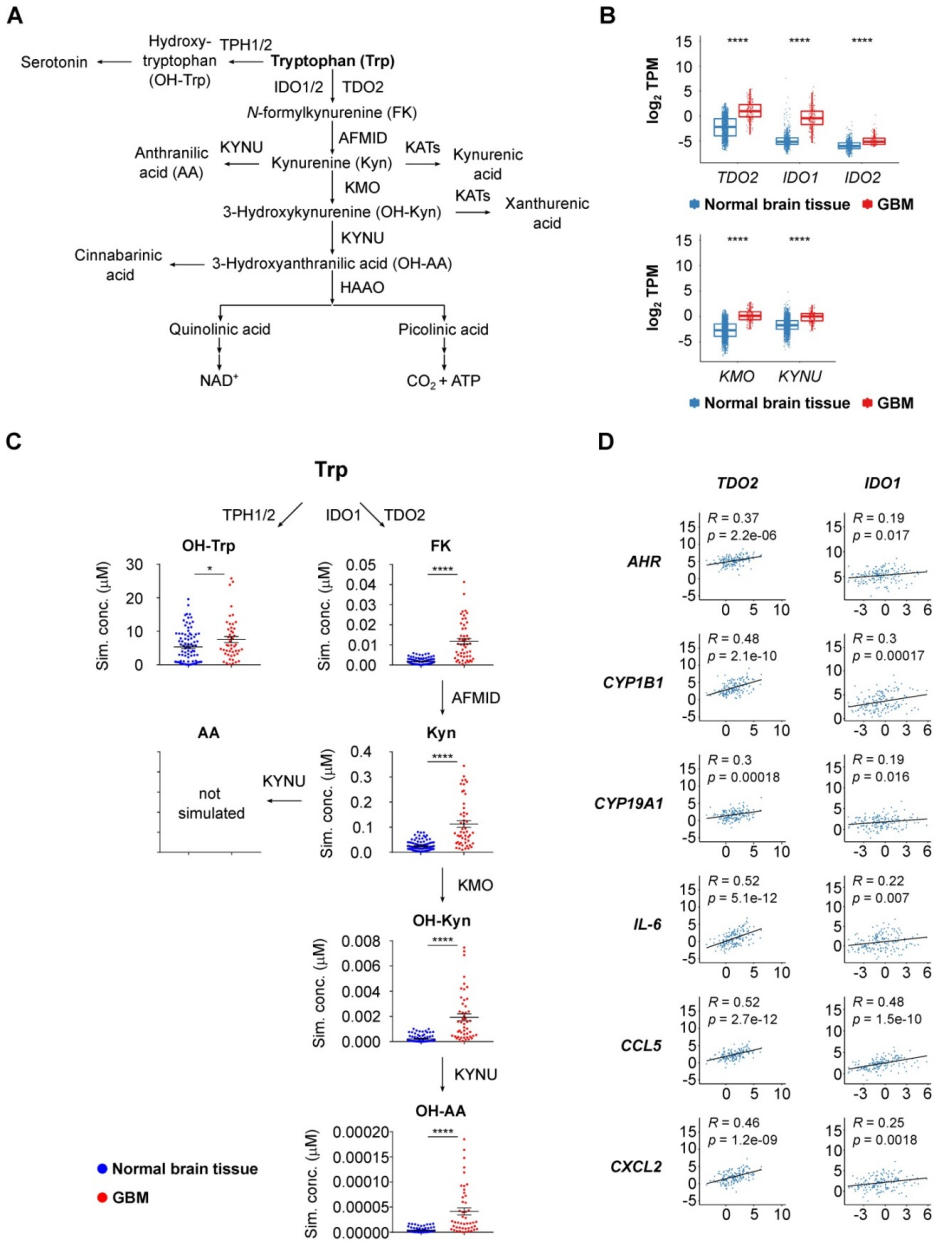
** Upregulated Trp metabolism correlates with AHR activity in glioblastoma. (A)** Schematic representation of Trp metabolism. **(B)** Boxplot representation of the expression of select Trp metabolism-associated enzymes in normal brain tissue (blue) (GTEx data) and in glioblastoma (GBM) tissue (red) (TCGA data) represented as log_2_ transcripts per million (log_2_ TPM) (Wilcoxon rank-sum test, **** *P* < 0.0001). **(C)** RNA-seq data from the GTEx and TCGA databases were integrated into a mathematical model of Trp metabolism to predict the metabolite concentrations in healthy brain or GBM tissue for: OH-Trp, FK, Kyn, OH-Kyn, OH-AA. Data are mean +/- SEM, outliers were excluded with ROUT. Data were analyzed with two-tailed unpaired Student's t test. *P < 0.05, ****P < 0.0001. **(D)** Pearson's correlation coefficient (R) estimated for the expression of the Trp-degrading enzymes *TDO2* or *IDO1*, and the expression of select AHR target genes in GBM tissue (TCGA data). P values are given as numbers. See also Figure S1. Abbreviations: AA: anthranilic acid; AFMID: arylformamidase; AHR: aryl hydrocarbon receptor; FK: N-formylkynurenine; GBM: glioblastoma; GTEx: Genotype-Tissue Expression; HAAO: 3-hydroxy-anthranilic acid-3,4-dioxygenase; IDO1: indoleamine-2,3-dioxygenase 1; KATs: kynurenine aminotransferases; KMO: kynurenine 3-monooxygenase; Kyn: kynurenine; KYNU: kynureninase; NAD+: nicotinamide adenine dinucleotide; OH-AA: hydroxy-anthranilic acid; OH-Kyn: hydroxy-kynurenine; OH-Trp: hydroxy-tryptophan; R: Pearson's correlation coefficient; RNA-seq: RNA-sequencing; ROUT: robust regression and outlier removal; SEM: Standard error of mean; Sim. conc.: simulated concentrations; TCA: trichloroacetic acid; TCGA: The Cancer Genome Atlas; TDO2: tryptophan-2,3-dioxygenase; TPH1/2: tryptophan hydroxylase 1/2; TPM: transcripts per million; Trp: tryptophan.

To obtain a first insight into the activity of Trp metabolism in glioblastoma, we analyzed enzyme expression and predicted metabolite abundance in glioblastoma tissue. We found an enhanced transcript expression of TCEs and KP-enzymes in glioblastoma tissue compared to healthy brain tissue (Figure 1A,B, Figure S1A). We next integrated the GTEx normal brain tissue and TCGA glioblastoma (GBM) gene expression data into an extended version of a previously published mathematical model of Trp metabolism that is based on existing kinetic data for the enzymatic conversions and transporters 64. In line with the results above, the mathematical modeling predicted increased levels of Trp-derived metabolites in glioblastoma tissue (Figure 1C). Trp catabolites activate the AHR and AHR activity can be assessed by induction of AHR target genes. The expression of select AHR target genes, including *AHR* itself, positively correlated with *TDO2* expression (Figure 1D) and to a lesser extent with *IDO1* expression (Figure 1D), pointing towards TCE-mediated AHR activation in glioblastoma.

### Multiplexed LC-MS/MS combined with isobaric labeling enables sensitive and high-throughput measurement of Trp and its metabolites in human serum

**Figure 2:**
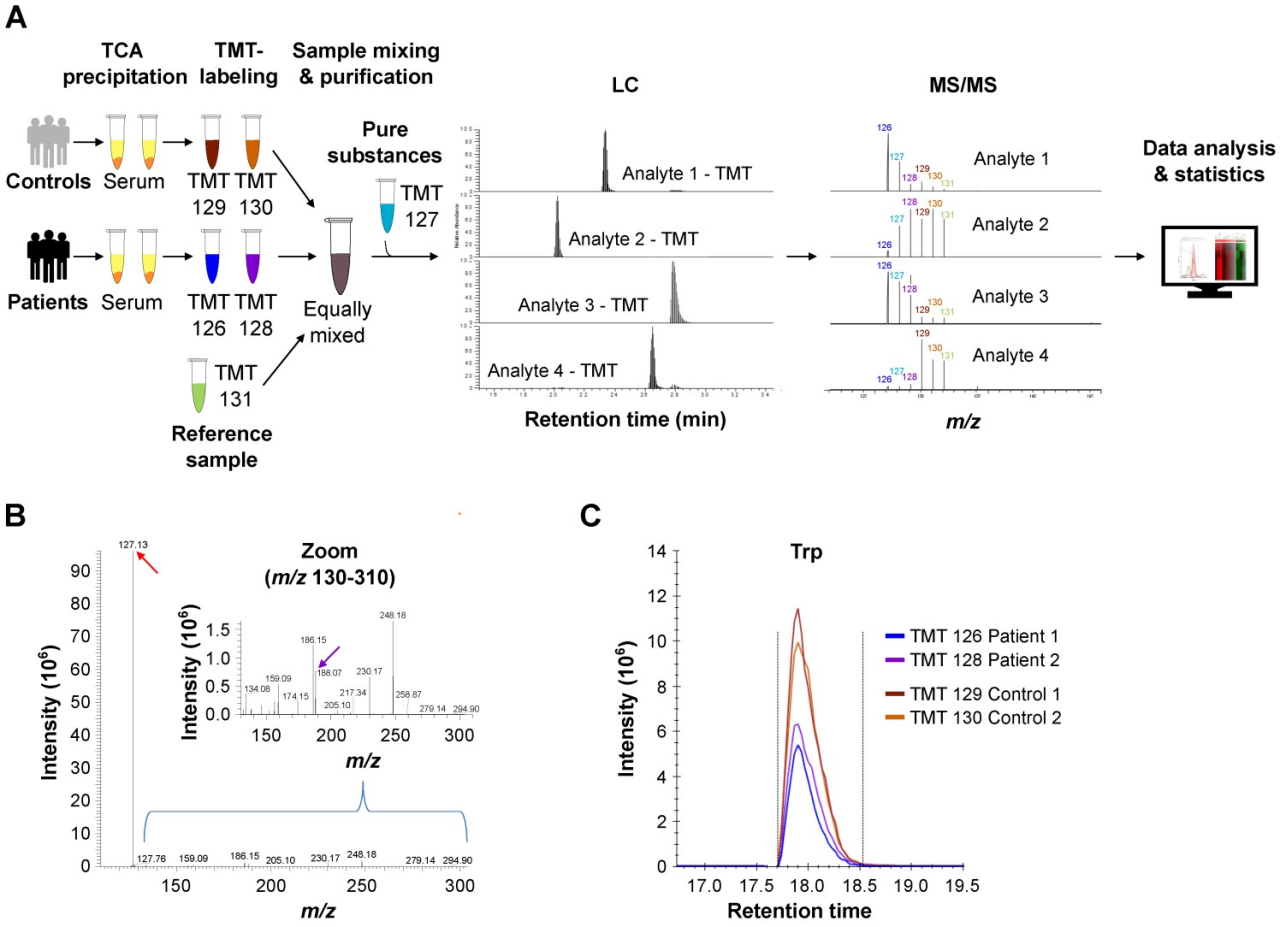
** Multiplex metabolite measurements applying tandem mass spectrometry in combination with isobaric chemical labeling enable efficient and sensitive measurement of Trp and its downstream metabolites in human sera. (A)** Workflow for multiplex measurements of Trp and its downstream metabolites in the sera of patients and controls in sixplexes by tandem mass spectrometry in combination with isobaric chemical labeling employing tandem mass tag (TMT®) reagents. Six individual samples, comprising two control serum samples, two glioblastoma patient samples, one reference serum sample and one sample with a mix of the pure substances, were labeled with six different TMT® reagents, mixed and analyzed by LC-MS/MS. For labeled analytes, reporter ion intensities reflect relative concentrations of the analytes. Spectrum processing was performed with Skyline. **(B)** Representative MS/MS-spectrum showing a higher TMT® reporter ion intensity (red arrow) in comparison to the (non-labeled) structural fragment intensities (exemplarily *m/z* 188.07 (violet arrow)) from L-Trp. **(C)** Representative image of the reporter fragment ion intensities of one plex showing the higher intensities of two control samples (orange and red line) in comparison to two patient samples (blue and violet line). See also Figure S1. Abbreviations: LC-MS/MS: liquid chromatography tandem mass spectrometry; TCA: trichloroacetic acid; TMT®: tandem mass tag; Trp: tryptophan.

To explore if also systemic Trp metabolism is altered in glioblastoma patients compared to healthy age- and sex-matched controls, we measured Trp and its derivatives in serum. To this end, we developed an innovative multiplexing approach based on the combination of LC-MS/MS with chemical isobaric labeling using tandem mass tags (TMT^®^) 79 (Figure S1B). The TMT^®^ reagent is composed of an amine-reactive NHS-ester group, a spacer arm and a mass reporter moiety. The different TMT^®^ reagents have the same mass and structure, but contain different numbers of heavy isotopes in the mass reporter region. The fragmentation of the TMT^®^-labeled analyte produces unique reporter ions, which are used for multiplexed sample quantitation. TMT^®^ reagents are commonly applied in proteomics 79 and have been used to measure amino acids in cell culture 80. To analyze Trp metabolism in human serum we adapted this approach for a multiplexed relative quantification of Trp and its amino group-containing metabolites, namely hydroxy (OH)-Trp, *N*-formylkynurenine (FK), Kyn, OH-Kyn, anthranilic acid (AA) and OH-AA. To compare metabolite levels in patients and controls, we measured sixplexes containing two patient and two control serum samples each, as well as a mixture of the pure compounds. In addition, a reference sample was included in each sixplex to enable comparison across different sixplexes. Each sample was labeled with one of six different TMT^®^ sixplex reagents. The mixture of these six samples was subjected to LC-MS/MS analysis, in which the analytes were separated by retention time and mass (Figure 2A). MS/MS fragmentation of the labeled analytes produced reporter ions that allowed for their relative quantitation in each of the six samples (Figure 2A). Of note, the TMT^®^ reporter ions show significantly higher intensities than the (non-labeled) structural fragments of the analyte (Figure 2B), proving that an efficient fragmentation is achieved and that the TMT^®^ reporter ions are ideal for quantitative purposes. This methodic setup further facilitated the distinction of metabolite levels in patient and control sera (Figure 2C). In conclusion, this novel approach enabled us to compare the levels of Trp and its metabolites in the sera of patients and healthy controls with high throughput, sensitivity and accuracy.

### The levels of Trp and its metabolites are decreased in the sera of patients with glioblastoma

**Figure 3:**
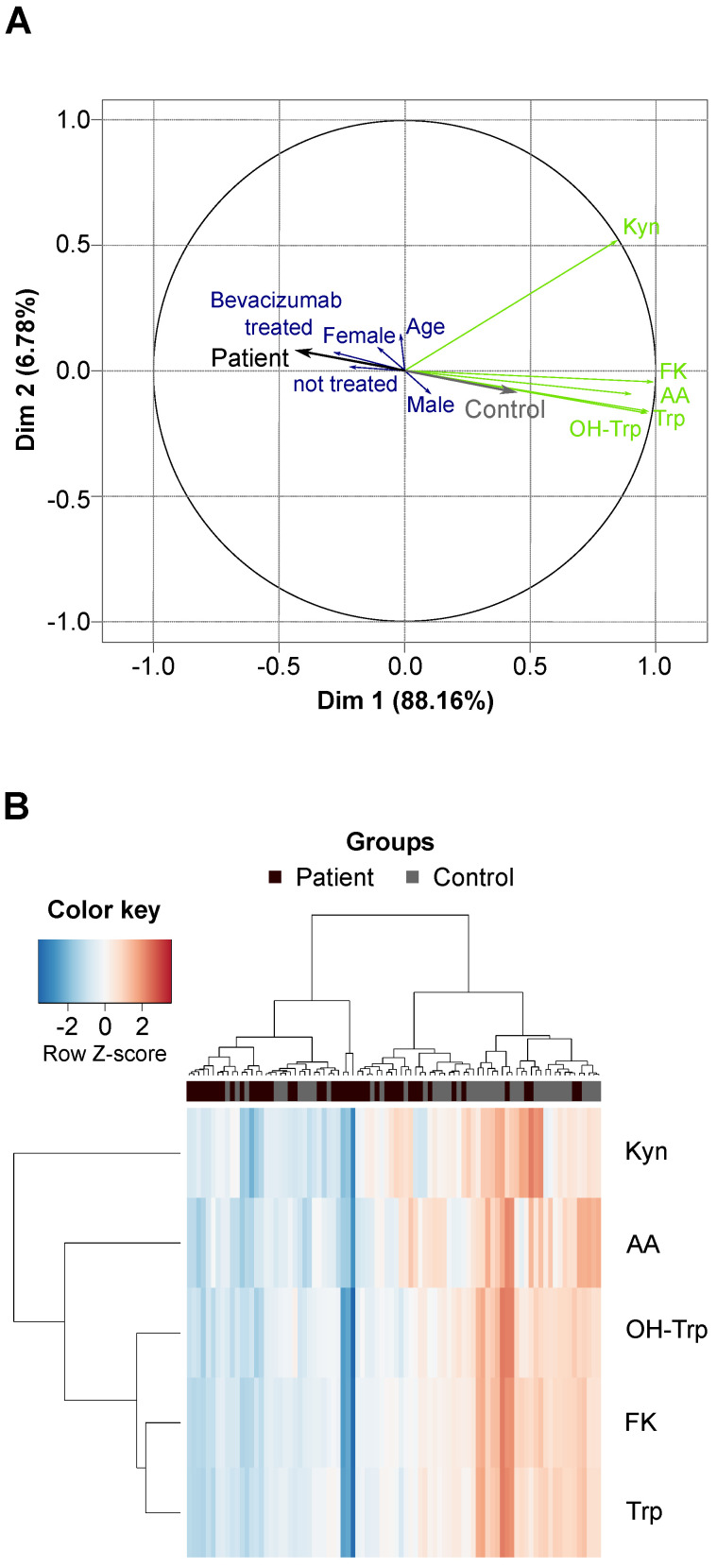
** Glioblastoma patients and healthy controls separate based on the serum levels of Trp and its metabolites. (A)** Principal component analysis bi-plot depicting the contribution of different factors to the variance of the metabolite measurement data. The influence of prior treatment with bevacizumab is depicted as “Bevacizumab treated” or “not treated”. **(B)** Unsupervised clustering of Trp and four Trp metabolites in patient (*n* = 43) and control (*n* = 43) serum samples. See also Figure S2, Table S9-S14. Abbreviation: AA: anthranilic acid; Dim: dimension; FK: *N*-formylkynurenine; Kyn: kynurenine; OH-Trp: hydroxy-tryptophan; Trp: tryptophan.

**Figure 4:**
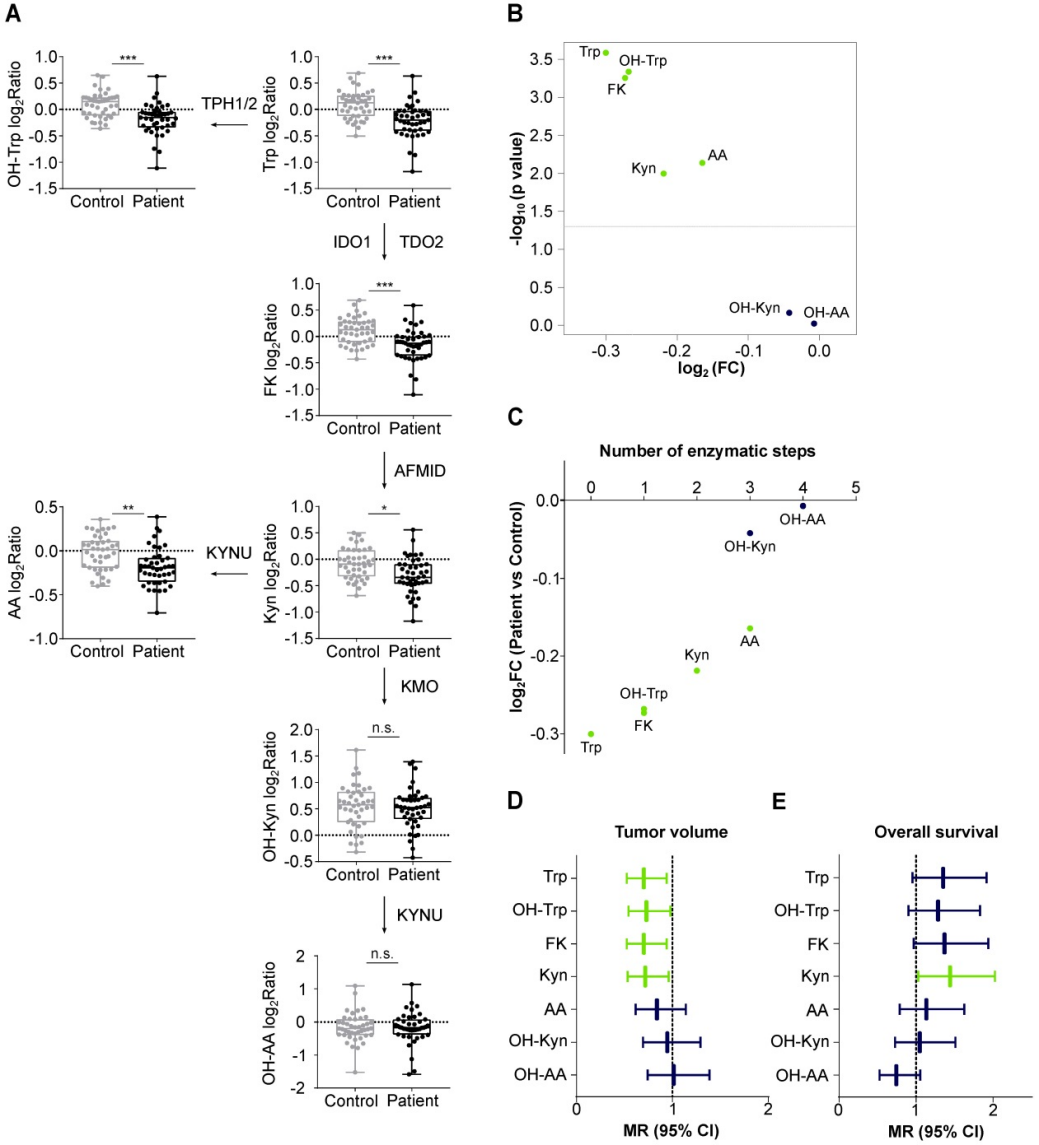
** Levels of Trp and its metabolites are decreased in the sera of glioblastoma patients and associate with tumor volume. (A)** Metabolite abundance of Trp, OH-Trp, FK, Kyn, AA, OH-Kyn, OH-AA in sera of age- and sex-matched controls (grey, *n* = 43) and glioblastoma patients (black, *n* = 43) relative to reference sample depicted as log2Ratio. Boxplots show median, 25^th^ and 75^th^ percentile, and whiskers maximal and minimal values. *P < 0.05, **P < 0.01, ***P < 0.001. n.s. not significant. **(B)** Volcano plot showing log_2_FC of metabolite levels (x-axis) in patient (*n* = 43) versus control (*n* = 43) samples and corresponding -log_10_ p values (y-axis). Metabolites highlighted in green were significantly decreased in patient versus control sera. **(C)** Same data depicted as in (B). Metabolite levels in patient versus control samples in log_2_FC (y-axis) are ordered according to enzymatic steps away from Trp (x-axis). Metabolites highlighted in green were significantly decreased in patient versus control sera. **(D)** Forest plot depicting the association of metabolite levels in peripheral blood of patients (*n* = 43) with tumor volume based on MRI analyses. Mean ratio (MR) and 95% confidence intervals (CI) are shown. Green bars represent significant relationships. **(E)** Forest plot depicting the association of metabolite levels in peripheral blood of patients (n = 32) with overall survival. Mean ratio (MR) and 95% confidence intervals (CI) are shown. Green bar represents significant relationship. See also Table S15. Abbreviations: AA: anthranilic acid; AFMID: arylformamidase; CI: confidence interval; FC: fold change; FK: *N*-formylkynurenine; IDO1: indoleamine-2,3-dioxygenase 1; KMO: kynurenine 3-monooxygenase; Kyn: kynurenine; KYNU: kynureninase; MR: mean ratio; MRI: magnetic resonance imaging; OH-AA: hydroxy-anthranilic acid; OH-Kyn: hydroxy-kynurenine; OH-Trp: hydroxy-tryptophan; TDO2: tryptophan-2,3-dioxygenase; TPH1/2: tryptophan hydroxylase 1/2; Trp: tryptophan.

We compared Trp and its metabolites in the sera of 43 patients with recurrent glioblastoma and 43 age- and sex-matched healthy controls (Table S9, Table S10). The high variation of the serum levels of Trp, OH-Trp, FK, Kyn and AA was the main driver of the difference between the patient and control cohorts, while other covariates (e.g. age, sex and prior bevacizumab treatment (bevacizumab treated/not treated)) showed minor contribution to the cohort differences (Figure 3A). An association analysis confirmed that prior bevacizumab intake (Table S11) and the cumulative dose of bevacizumab prior to the study blood draw did not affect metabolite levels (Figure S2). Also, MGMT promotor methylation status, EGFR amplification and PTEN loss showed no effect on metabolite levels (Table S12-S14). Clustering of the sera revealed that the majority of glioblastoma patients displayed lower metabolite levels than the controls (Figure 3B). Only few patients displayed a metabolite profile that clustered better with control profiles, which generally showed slightly higher metabolite levels (Figure 3B). We had expected a decrease of Trp, but an increase of its metabolites. Surprisingly, however, the levels not only of Trp but also of its metabolites OH-Trp, FK, Kyn and AA were significantly lower in patients' sera than in control sera (Figure 3B, Figure 4A,B). Trp levels showed the highest reduction in patients compared to control sera. The more enzymatic steps there were between the metabolites and Trp, the smaller the decrease in the metabolite levels in the patient sera became (Figure 4C, Table S15) pointing towards a prominent role of Trp in controlling the systemic levels of its metabolites. Next, we asked whether the tumor volume affects the systemic levels of Trp and its metabolites. Trp, OH-Trp, FK and Kyn levels in the peripheral blood showed a negative relationship with the tumor volumes determined by MRI analyses (Figure 4D), indicating that a higher tumor volume associated with reduced systemic metabolite levels. In addition, high Kyn levels associated with increased overall survival and there was also a trend for high Trp, OH-Trp and FK levels to associate with better survival (Figure 4E).

### Visualization of the spatial distribution of Trp and its direct metabolite FK in glioblastoma

We employed MALDI MSI to obtain spatial information of the distribution of Trp and its direct downstream metabolite FK in 17 glioblastoma tissue sections. The median age of the glioblastoma patients (*n* = 6 female, *n* = 11 males) was 60.82 years and the molecular characteristics of their tumors are shown in Table S16.

**Figure 5:**
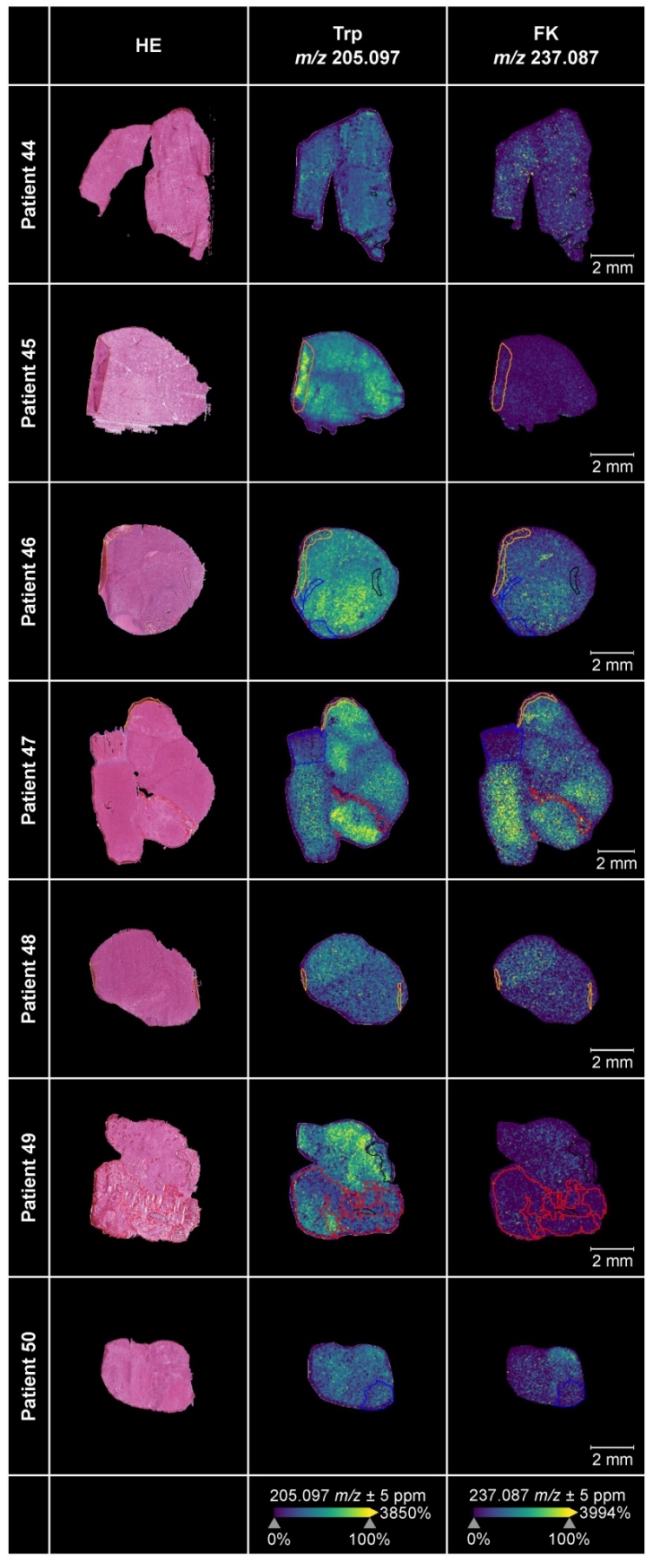
** Trp and its metabolite FK in glioblastoma tumor tissue.** MALDI MSI of Trp and FK distribution in human glioblastoma samples (middle and right column) and corresponding annotated HE-stained adjacent tissue sections (left column) (*n* = 7). Displayed are Trp-D5 normalized ion density maps of Trp (*m/z* 205.097) and FK (*m/z* 237.087). Trp and FK were measured using a FT-ICR MS in positive ion mode at a raster size of 50 µm. Annotations were drawn in SCiLS Lab; black: necrosis; blue: infiltration zone; red: highly vascularized tumor tissue or blood; yellow: artefacts. See also Figure S3, Table S16. Abbreviations: FK: *N*-formylkynurenine; FT-ICR MS: Fourier-transform ion cyclotron resonance mass spectrometer; HE: hematoxylin-and-eosin; MSI: mass spectrometry imaging; Trp: tryptophan; Trp-D5: deuterated tryptophan.

For ten of these glioblastomas adjacent sections were available, which we stained with hematoxylin-and-eosin (HE). Projection of different areas identified by HE onto the tissue section used for MALDI MSI was possible in seven samples (Figure 5). This enabled us to confirm that we were indeed measuring vital glioblastoma tissue. Only small regions of the seven glioblastoma sections contained hypervascularized tumor tissue or blood, necrosis, infiltration zone or preparation artefacts. The sections in which projection of different areas was not feasible or the ones without adjacent sections are shown in Figure S3A-S3B. The annotations showed that Trp and its direct downstream metabolite FK, which we used as a marker for Trp catabolism down the KP, were highest in vital tumor tissue as necrotic or hypervascularized tumor tissue areas showed less abundance. Trp was present in all of the glioblastoma tissue samples. Further, many of the glioblastomas metabolized Trp to produce downstream FK. In these samples, Trp was not markedly reduced or consumed but still detectable. In other samples, despite the presence of Trp, hardly any FK was measurable. This hints to differences between glioblastomas that either metabolize Trp to FK or not.

### Trp metabolism and AHR activation are present in almost all the cell types in glioblastoma tissue

**Figure 6:**
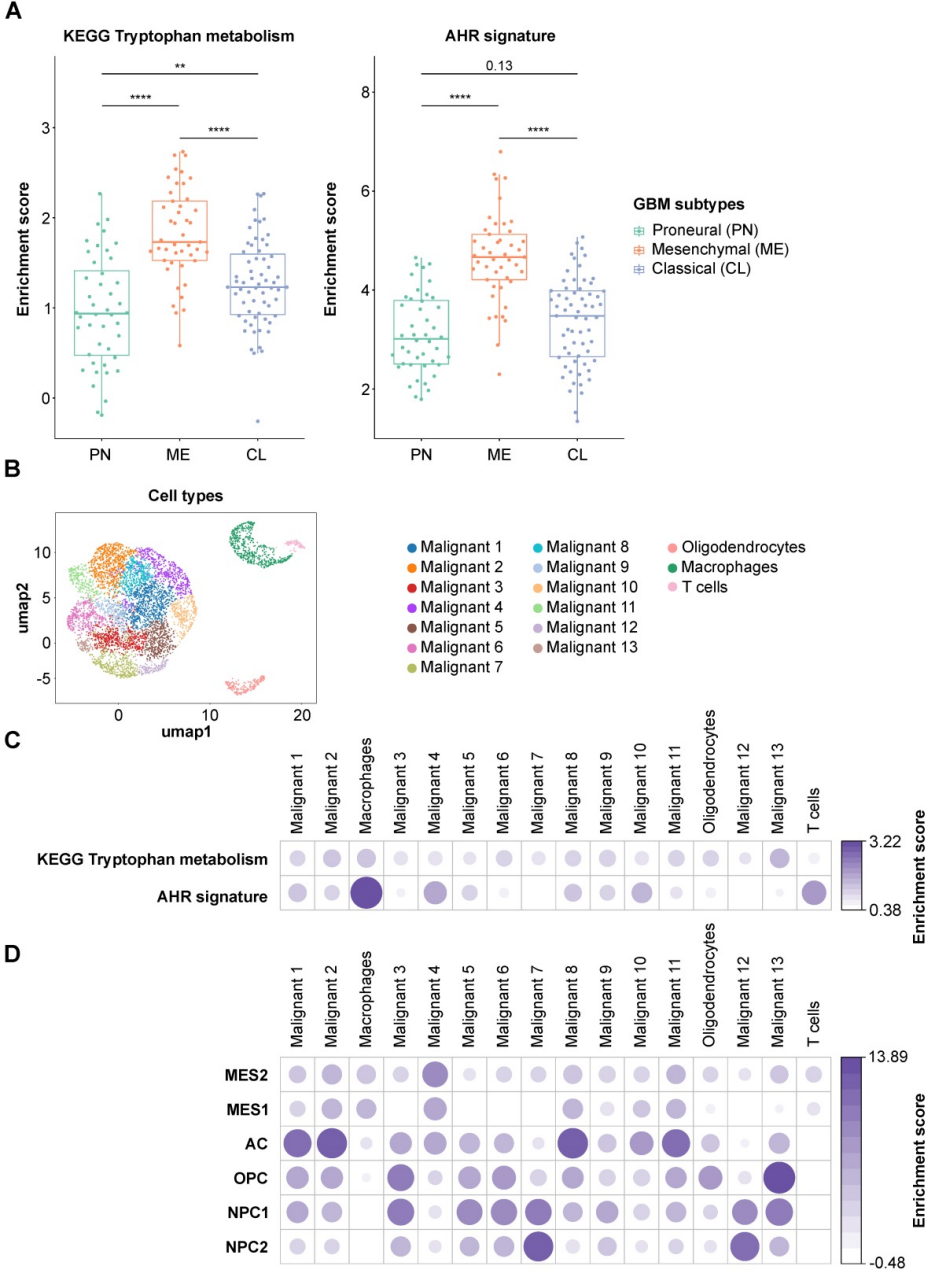
** Trp metabolism and AHR activity are upregulated in tumor cells and in immune cells in glioblastoma. (A)** Boxplot representation of the biological process activity (BPA) score in TCGA glioblastoma (GBM) data across the three transcriptional subtypes defined by Wang et al. 8 using gene sets of the KEGG tryptophan metabolic pathway (left) and the AHR activation signature (right) 43. Group comparisons were performed using Wilcoxon rank-sum test, p values are given as numbers or **P < 0.01, ****P < 0.0001. **(B)** UMAP representation of the Louvain-clustered cell populations from the scRNA-seq dataset (GSE131928). The scatter shows the different malignant cell clusters and three distinct non-malignant clusters, namely oligodendrocytes, macrophages and T cells. **(C)** Bubble heatmap representation of the median BPA score of the Louvain clusters defined in (B) for the KEGG tryptophan metabolism pathway and the AHR signature as in (A). **(D)** Bubble heatmap representation of the median BPA score of the Louvain clusters defined in (B) for the six different cell states of glioblastoma cells defined by Neftel et al. 7: MES1/2, AC, OPC, NPC1/2. See also Figure S4. Abbreviations: AC: astrocyte-like; AHR: aryl hydrocarbon receptor; BPA: biological process activity; CL: classical; KEGG: Kyoto Encyclopedia of Genes and Genomes; Kyn: kynurenine; ME: mesenchymal; MES1/2: mesenchymal-like 1/2; NPC1/2: neural progenitor cell-like 1/2; OH-Kyn: hydroxy-kynurenine; OPC: oligodendrocytic precursor cell-like; PN: proneural; scRNA-seq: single cell RNA-sequencing; TCGA: The Cancer Genome Atlas; Trp: tryptophan; UMAP: uniform manifold approximation and projection.

To assess the difference in Trp metabolism across glioblastoma subtypes, we analyzed the TCGA GBM RNA-seq expression data. The patient samples were grouped according to the transcriptional subtypes described by Wang et al. 8. Using the KEGG 59 Trp metabolism genes, we generated a per-sample enrichment score reflecting the biological activity of the Trp metabolic pathway in the different glioblastoma subtypes. The mesenchymal subtype showed the highest enrichment of Trp metabolism while the proneural subtype displayed the lowest enrichment scores (Figure 6A, left). In agreement with a role of Trp metabolism in AHR activation, AHR activity, assessed by a pan-tissue signature based on AHR target gene expression 43, was also highest in the mesenchymal subtype (Figure 6A, right). As the mesenchymal subtype is highly infiltrated with immune cells, in particular myeloid cells including microglia and macrophages 81, we went on to analyze which cell types contribute to Trp metabolism and AHR activity in a publicly available scRNA-seq dataset of glioblastoma tissue 7. The UMAP representation showed four distinct cell populations. Using specific marker genes, we characterized the distinct populations as malignant tumor cells, oligodendrocytes, macrophages and T cells (Figure 6B, Figure S4A). The macrophages present in the dataset primarily showed a strong M2 signature enrichment, almost no M1 signature enrichment and some M0 signature enrichment (Figure S4B). All cell populations present in glioblastoma tissue showed an enrichment of Trp metabolism (Figure 6C). Moreover, in particular macrophages and T cells, but also oligodendrocytes and most of the malignant tumor cell sub-populations showed AHR activation (Figure 6C). Next, we characterized the cell populations according to the cell states defined by Neftel et al. 7: mesenchymal-like 1 and 2 (MES1/2), astrocyte-like (AC), oligodendrocytic precursor cell-like (OPC) and neural progenitor cell-like 1 and 2 (NPC1/2). Louvain clusters representing the sub-populations of malignant cells enriched with markers for MES1/2 and AC subsets showed rather high AHR activity (e.g. malignant cluster 4 and 10), while malignant cell clusters enriched with markers for NPC1/2 subsets showed no AHR activity (malignant cluster 7 and 12) (Figure 6D). Taken together, Trp metabolism and AHR activation are enriched in most cell populations present in glioblastoma tissue, with a particularly pronounced AHR activation in immune cells. Finally, analysis of the effect of AHR activation on overall survival using the GBM TCGA data revealed that glioblastoma patients showing high AHR activity are at a twofold higher risk of worse overall survival (Table S17).

## Discussion

Immune suppression and escape are increasingly recognized as critical traits of malignancy 82. During cancer progression, Trp metabolism may represent an important pathway for immune escape, while also promoting the malignant phenotype of cancer cells in an autocrine fashion. The increasing interest in Trp metabolism, which in addition to promoting cancer is involved in controlling autoimmune diseases, immune tolerance and chronic infection 23, prompted us to develop a method for the multiplexed measurement of Trp and its metabolites using tandem mass spectrometry in combination with chemical isobaric labeling using TMT^®^ reagents 79 (Figure 2). Chemical isobaric labeling enabled us to measure multiple amino group-bearing metabolites simultaneously in six samples with high sensitivity, accuracy and efficiency (Figure 2). One key advantage of isobaric labeling over a label-free workflow is the capability of multiplexing and thus preparing and measuring multiple samples simultaneously. Measuring multiple samples in one LC-MS/MS run excludes run-to-run variation for the samples measured and also reduces the number of datasets that need to be processed. Generally, multiplexing reduces sample processing variability because from the moment when samples are mixed the quantitative pattern is conserved as all (unavoidable) variations in steps like e.g. purification, fractionation, solving, loading, and LC-separation affect the mixed samples equally. The current available isobaric chemical tags facilitate the simultaneous analysis of up to 18 experimental samples 83, which significantly enhances throughput and reduces turnaround time. Moreover, isobaric labeling can be easily adapted for high-throughput measurements as sample preparation can be parallelized and automated. Furthermore, absolute quantitation of Trp and its metabolites could be achieved by calibration with internal standards.

Analysis of glioblastoma sera using this novel multiplex method revealed that in patients with glioblastoma systemic Trp availability is limited to a degree that leads to a reduction of its metabolites. Of note, we were able to measure reduced levels of Trp and its metabolites in the sera of glioblastoma patients compared to healthy controls (Figure 3, Figure 4), despite the fact that blood sampling from neither was controlled for time of blood draw or fasting before sampling. Hence, the observed effects appear to be rather robust and stable. Among the metabolites analyzed, Trp was most strongly reduced in the sera of glioblastoma patients. The low Trp levels in glioblastoma patients could potentially contribute to the poor response of glioblastoma patients to immune checkpoint blockade 84 as a recent study in mice showed that low systemic Trp levels decreased efficacy of anti-cancer therapy with immune checkpoint inhibitors 85. Moreover, the decrease of the Trp metabolites was stronger the less enzymatic steps separated them from Trp (Figure 4C), suggesting that the availability of Trp as an enzyme substrate determined the systemic levels of its metabolites. While a reduction in Trp has previously been reported in the blood of patients with glioblastoma 27, 86, 87, only the analysis of Trp in combination with multiple of its metabolites enabled us to identify Trp as the limiting factor for the systemic levels of its metabolites in glioblastoma patients. This underscores the advantage of analyzing multiple constituents of a metabolic pathway rather than single metabolites.

Before performing this study, we had anticipated systemic Kyn levels to be a surrogate marker for high Kyn levels in the tumor. However, our results revealed that systemic Trp metabolites do not appear to derive from the tumor but that systemic Trp levels control the levels of its downstream metabolites including Kyn in the periphery. This may explain why high systemic Kyn levels associated with better overall survival in our glioblastoma patient cohort (Figure 4E). Moreover, regression analysis of tumor volumes and metabolite levels of glioblastoma patients revealed a negative correlation between these two parameters, indicating that larger tumors are associated with a stronger systemic reduction in Trp and its metabolites (Figure 4D). This is in line with the observation that Trp and Kyn levels increase after surgery in glioblastoma patients 86 and suggests a link between the tumor and systemic metabolite levels. Measurement of Trp and its metabolites could be of clinical interest if their systemic levels enabled the selection and monitoring of patients for treatment with drugs interfering with Trp catabolism or AHR activation. By enabling the precise comparison of Trp and its metabolites in different samples, while being time efficient with up to 18 samples measurable in a single experiment, the novel Trp multiplex method we developed would be ideally suited for this application.

Using MALDI MSI we spatially visualized the distribution of Trp and FK in glioblastoma tissue. Trp was present in all glioblastoma sections, and its direct metabolite FK was detectable in many but not all glioblastomas (Figure 5, Figure S3). Trp was not reduced in glioblastomas that degraded Trp to FK, while in other samples, despite the presence of Trp, hardly any FK was measurable. This heterogeneity, that may also underlie the outliers observed in our serum measurements (Figure 3), prompted us to analyze Trp metabolism across different types of glioblastoma. As we initially had observed an association between the expression of Trp-degrading enzymes and AHR activity, we made use of the pan-tissue AHR signature we previously developed 43 to also assess AHR activity. Confirming and expanding the results of others and us 25, 29, 88, we found that in addition to Trp metabolism also AHR activity is highest in the mesenchymal subtype of glioblastoma (Figure 6A). The increased activity of AHR in mesenchymal glioblastoma is of particular interest, as this subtype does not differ in AHR expression from the other glioblastoma subtypes 89, suggesting that it is not the abundance of the transcription factor, but that of its potentially Trp-derived agonists that leads to the increase in AHR activity in mesenchymal glioblastoma. As mesenchymal glioblastoma is characterized by high infiltration with immune cells, we went on to analyze publicly available single cell RNA-seq data of glioblastoma 7 for Trp metabolism and AHR activity in order to identify the cell types contributing to these processes. Our analysis of this dataset revealed that all the cell types identified in glioblastoma in the single cell analysis showed enriched Trp metabolism, including immune cells, namely macrophages and T cells, oligodendrocytes as well as all the malignant cell clusters (Figure 6C). These results suggested that Trp metabolism could activate the AHR in an autocrine or paracrine manner. However, the presence of transcripts of Trp-metabolizing enzymes does not indicate if sufficient concentrations of metabolites accumulate to activate the AHR. AHR activity in glioblastoma was upregulated particularly in macrophages and T cells, but to a lesser extent also in oligodendrocytes and all but two malignant cell clusters. In agreement with the results of the bulk RNA-seq data, Louvain clusters of the malignant cells with high enrichment for astrocyte-like markers and mesenchymal-like markers in the scRNA-seq dataset were the ones that showed high AHR activity, while in those enriched for neural progenitor cell-like markers AHR activity was absent (Figure 6D). As AHR activity in tumor-associated macrophages and in T cells has previously been shown to promote tumor progression by diverse mechanisms including the upregulation of adenosine production 89 and immune checkpoint molecule expression 90, these results suggest that Trp metabolism and AHR activation in glioblastoma may contribute to glioblastoma immune evasion. However, AHR activation does not always necessarily promote cancer as it also exerts tumor-suppressive effects and has been shown to inhibit tumor formation 91-93 and metastasis 94-96. These divergent effects of the AHR in cancer likely stem from the complexity of its activation and effects, which are cell type-, ligand- and context-specific 43, 97.

In conclusion, the novel techniques we developed to characterize Trp metabolism in biological fluids as well as tumor tissue could support the identification and monitoring of patients that may benefit from therapies altering the generation of Trp metabolites or AHR activation.

## Supplementary Material

Supplementary figures and tables.

## Abbreviations

AA: anthranilic acid
AC: astrocyte-like
ACN: acetonitrile
AFMID: arylformamidase
AHR: aryl hydrocarbon receptor
ANN: artificial neural network
BPA: biological process activity
CCBL: cysteine conjugate beta lyase
CE: collision energy
CI: confidence intervals
CL: classical
Coef.: coefficient
CV: coefficient of variation
DHB: 2,5-dihydroxybenzoic acid
Dim: dimension
EGFR: epidermal growth factor receptor
F: female
FA: formic acid
FC: fold change
FK: *N*-formylkynurenine
FLAIR: fluid attenuated inversion recovery
FPKM: fragments per kilobase of transcript per million mapped reads
FT-ICR: Fourier-transform ion cyclotron resonance
FWHM: full width at half maximum
GBM: glioblastoma
GEO: Gene Expression Omnibus
GTEx: Genotype-Tissue Expression
HAAO: 3-hydroxy-anthranilic acid-3,4-dioxygenase
HE: hematoxylin-and-eosin
HVGs: highly variable genes
ICB: immune checkpoint blockade
IDH: isocitrate dehydrogenase
IDO1/2: indoleamine-2,3-dioxygenase 1 and 2
KATs: kynurenine aminotransferases
KEGG: Kyoto Encyclopedia of Genes and Genomes
KMO: kynurenine 3-monooxygenase
KP: kynurenine pathway
Kyn: kynurenine
KYNU: kynureninase
LC: liquid chromatography
M: male
ME: mesenchymal
MES1/2: mesenchymal-like 1 and 2
MGMT: O^6^-methylguanine-DNA methyltransferase
MR: mean ratio
MS: mass spectrometry
MSI: mass spectrometry imaging
MS/MS: tandem mass spectrometry
n.a.: not applicable, clinical data not available
NAD^+^: nicotinamide adenine dinucleotide
NaOH: sodium hydroxide
NPC1/2: neural progenitor cell-like 1 and 2
OH-AA: hydroxy-anthranilic acid
OH-Kyn: hydroxy-kynurenine
OH-Trp: hydroxy-tryptophan
OPC: oligodendrocytic precursor cell-like
PAGA: partition-based graph abstraction
PN: proneural
PTEN: phosphatase and tensin homolog
R: Pearson's correlation coefficient
RNA-seq: RNA-sequencing
scRNA-seq: single cell RNA-sequencing
ROUT: robust regression and outlier removal
RT: radiotherapy
SD: standard deviation
SE: standard error
Se.coef.: standard error of the coefficient
SEM: standard error of mean
Sim. conc.: simulated concentrations
TCA: trichloroacetic acid
TCEs: tryptophan-catabolic enzymes
TCGA: The Cancer Genome Atlas
TDO2: tryptophan-2,3-dioxygenase
TEAB: triethylammonium bicarbonate
TFA: trifluoroacetic acid
TME: tumor microenvironment
TMM: trimmed mean of M values
TMT^®^: tandem mass tag
TMZ: temozolomide
TPH1/2: tryptophan hydroxylase 1/2
TPM: transcripts per million
Trp: tryptophan
Trp-D5: deuterated tryptophan
UMAP: uniform manifold approximation and projection
vs: versus
WT: wild type
Z: Wald test z score, which is the coefficient divided by its standard error
