# Supplementary material for: Tryptophan metabolism is inversely regulated in the tumor and blood of patients with glioblastoma
Source: Theranostics. 2021 Sep 3;11(19):9217–33. doi: 10.7150/thno.60679 (PMC8490504; doi:10.7150/thno.60679)
Supplement: Supplementary file 1 — Supplementary figures and tables. [file thnov11p9217s1.pdf]

Supplementary Material

Tryptophan metabolism is inversely regulated in the tumor and blood of patients with glioblastoma

Verena Panitz[1,2,*], Saša Končarević[3,*], Ahmed Sadik[1,4,*], Dennis Friedel[1,4], Tobias Bausbacher[5], Saskia Trump[6], Vadim Farztdinov[3,**], Sandra Schulz[5], Philipp Sievers[7,8], Stefan Schmidt[5], Ina Jürgenson[1,2], Stephan Jung[3], Karsten Kuhn[3], Irada Pflüger[9], Suraj Sharma[10], Antje Wick[2], Pauline Pfänder[1,4], Stefan Selzer[3], Philipp Vollmuth[9], Felix Sahm[7,8], Andreas von Deimling[7,8], Ines Heiland[10], Carsten Hopf[5], Peter Schulz-Knappe[3,***], Ian Pike[11], Michael Platten[12,13], Wolfgang Wick[2,14], Christiane A. Opitz[1,2,§]

[1]DKTK Brain Cancer Metabolism Group, German Cancer Research Center (DKFZ), 69120 Heidelberg, Germany.

[2]Department of Neurology and National Center for Tumor Diseases, Heidelberg University Hospital, 69120 Heidelberg, Germany.

[3]Proteome Sciences R&D GmbH & Co. KG, Altenhöferallee 3, 60438 Frankfurt/Main, Germany.

[4]Faculty of Bioscience, Heidelberg University, 69120 Heidelberg, Germany.

[5]Center for Mass Spectrometry and Optical Spectroscopy (CeMOS), Mannheim University of Applied Sciences, 68163 Mannheim, Germany.

[6]Molecular Epidemiology Unit, Berlin Institute of Health at Charité - Universitätsmedizin Berlin, 10117 Berlin, Germany.

[7]Department of Neuropathology, Institute of Pathology, Heidelberg University Hospital, 69120 Heidelberg, Germany.

[8]Clinical Cooperation Unit Neuropathology, German Consortium for Translational Cancer Research (DKTK), German Cancer Research Center (DKFZ), 69120 Heidelberg, Germany.

[9]Department of Neuroradiology, Heidelberg University Hospital, 69120 Heidelberg, Germany.

[10]Department of Arctic and Marine Biology, UiT, The Arctic University of Norway, 9037 Tromsø, Norway.

[11]Proteome Sciences plc, 5 Dashwood Lang Road, Bourne Business Park, Addlestone, Surrey KT15 2HJ, United Kingdom.

[12]Department of Neurology, Medical Faculty Mannheim, University of Heidelberg, 68167 Mannheim, Germany.

[13]DKTK Clinical Cooperation Unit Neuroimmunology and Brain Tumor Immunology, German Cancer Research Center (DKFZ), 69120 Heidelberg, Germany.

[14]Clinical Cooperation Unit Neurooncology, German Cancer Research Center (DKFZ), 69120 Heidelberg, Germany.

*These authors contributed equally

[§]Corresponding author: c.opitz@dkfz.de

**Current address: Core Facility - High-Throughput Mass Spectrometry, Charité - Universitätsmedizin Berlin, corporate member of Freie Universität Berlin, Humboldt-Universität zu Berlin and Berlin Institute of Health (BIH), 10117 Berlin, Germany.

***Current address: Mittelorbroich 125, 47839 Krefeld, Germany.

Figure S1

A

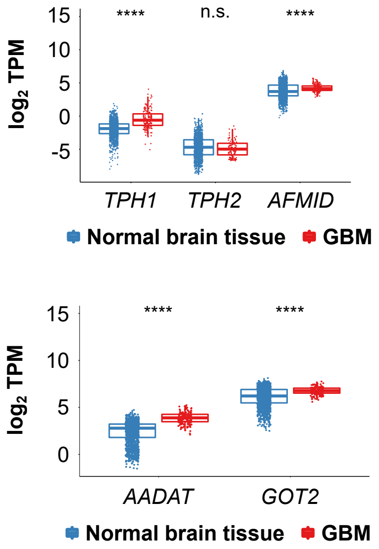

15
10
5
0
-5
log2 TPM
****
n.s.
****
TPH1
TPH2
AFMID
Normal brain tissue
GBM
15
10
5
0
log2 TPM
****
****
AADAT
GOT2
Normal brain tissue
GBM

B

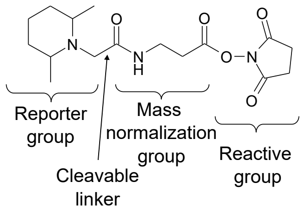

N
O
N
H
O
O–N
O
O
Reporter group
Mass normalization group
Cleavable linker
Reactive group

Figure S1. Trp metabolism-associated genes are upregulated in glioblastoma, related to Figures 1 and 2.

(A) Boxplot representation of the expression of select Trp metabolism-associated enzymes in normal brain tissue (blue) (GTEx data) and in glioblastoma (GBM) tissue (red) (TCGA data) represented as $\log_2$ transcripts per million ($\log_2$ TPM) (Wilcoxon rank-sum test, **** $P < 0.0001$, n.s. not significant). CCBL1 (KAT I) and CCBL2 (KAT III) were not expressed in the TCGA data.

(B) Chemical structure of TMT® reagents.

Abbreviations: AADAT: alpha-aminoadipate aminotransferase; AFMID: arylformamidase; CCBL: cysteine conjugate beta lyase; GBM: glioblastoma; GOT2: glutamate oxaloacetate transaminase 2; GTEx: Genotype-Tissue Expression; KAT: kynurenine aminotransferase; TCGA: The Cancer Genome Atlas; TMT®: tandem mass tag; TPH1/2: tryptophan hydroxylase 1/2; TPM: transcripts per million; Trp: tryptophan.

Figure S2

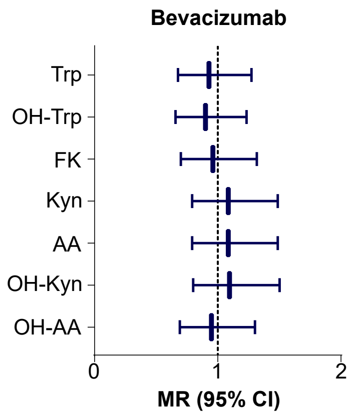

Figure S2. Bevacizumab treatment does not influence metabolite levels in peripheral blood of patients, related to Figure 3.

Forest plot depicting the association of metabolite levels in peripheral blood of patients (*n* = 42) with the cumulative dose of bevacizumab received prior to blood draw. Mean ratio (MR) and 95% confidence intervals (CI) are shown.

See also Table S11.

Abbreviations: AA: anthranilic acid; CI: confidence interval; FK: *N*-formylkynurenine; Kyn: kynurenine; MR: mean ratio; OH-AA: hydroxy-anthranilic acid; OH-Kyn: hydroxy-kynurenine; OH-Trp: hydroxy-tryptophan; Trp: tryptophan.

Figure S3

A

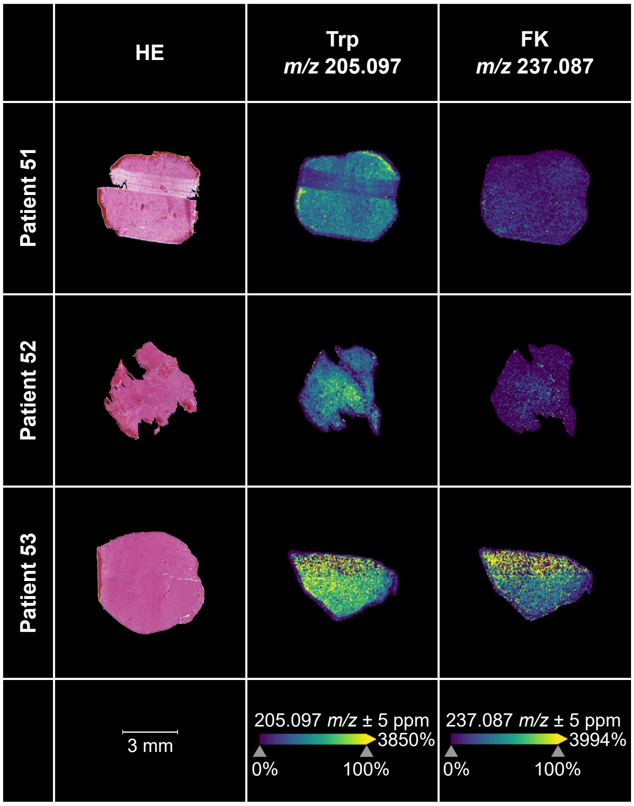
HE
Trp
m/z 205.097
FK
m/z 237.087
Patient 51
Patient 52
Patient 53
3 mm
205.097 m/z ± 5 ppm
3850%
0%
100%
237.087 m/z ± 5 ppm
3994%
0%
100%

B

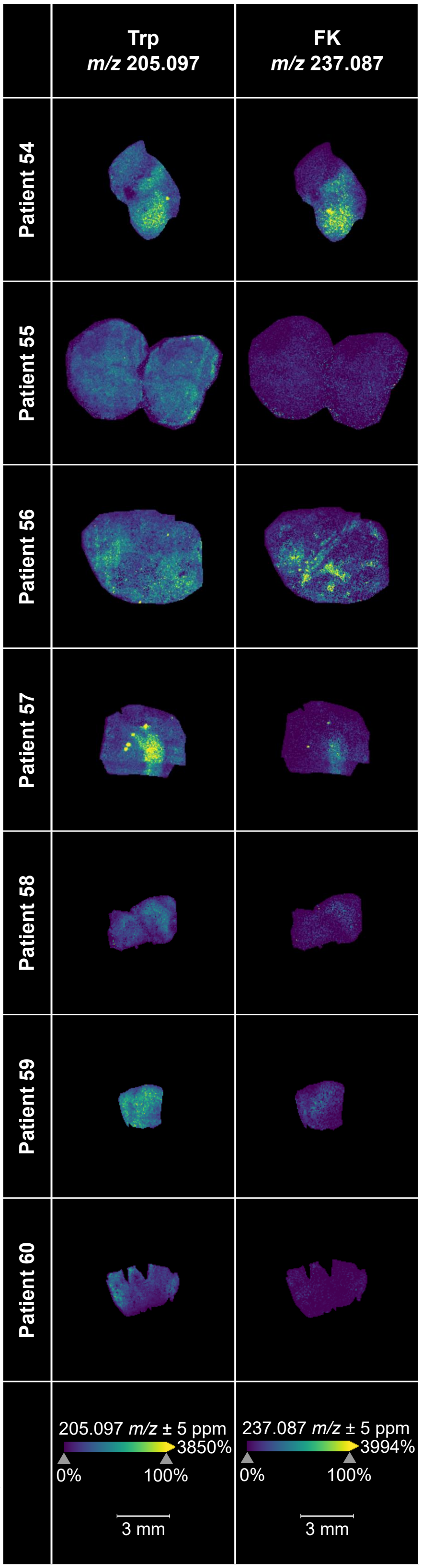
Trp
m/z 205.097
FK
m/z 237.087
Patient 54
Patient 55
Patient 56
Patient 57
Patient 58
Patient 59
Patient 60
205.097 m/z ± 5 ppm
3850%
0%
100%
237.087 m/z ± 5 ppm
3994%
0%
100%
3 mm
3 mm

Figure S3. Trp and its metabolite FK in glioblastoma tumor tissue, related to Figure 5.

(A) MALDI MSI of Trp and FK distribution in human glioblastoma samples (middle and right column) and corresponding annotated HE-stained adjacent tissue sections (left column) (n = 3). Displayed are Trp-D5 normalized ion density maps of Trp (*m/z* 205.097) and FK (*m/z* 237.087). Trp and FK were measured using a FT-ICR MS in positive ion mode at a raster size of 50 µm. Slides shown contain preparation artefacts. Annotations of HE-stainings: black: necrosis; red: highly vascularized tumor tissue or blood; yellow: artefacts.

(B) MALDI MSI of Trp and FK distribution in human glioblastoma samples (n = 7). Displayed are Trp-D5 normalized ion density maps of Trp (*m/z* 205.097) and FK (*m/z* 237.087). Trp and FK were measured using a FT-ICR MS in positive ion mode at a raster size of 50 µm. Samples without available adjacent HE-stained slide are shown.

See also Table S16.

Abbreviations: FK: *N*-formylkynurenine; FT-ICR MS: Fourier-transform ion cyclotron resonance mass spectrometer; HE: hematoxylin-and-eosin; MSI: mass spectrometry imaging; Trp: tryptophan; Trp-D5: deuterated tryptophan.

Figure S4

A

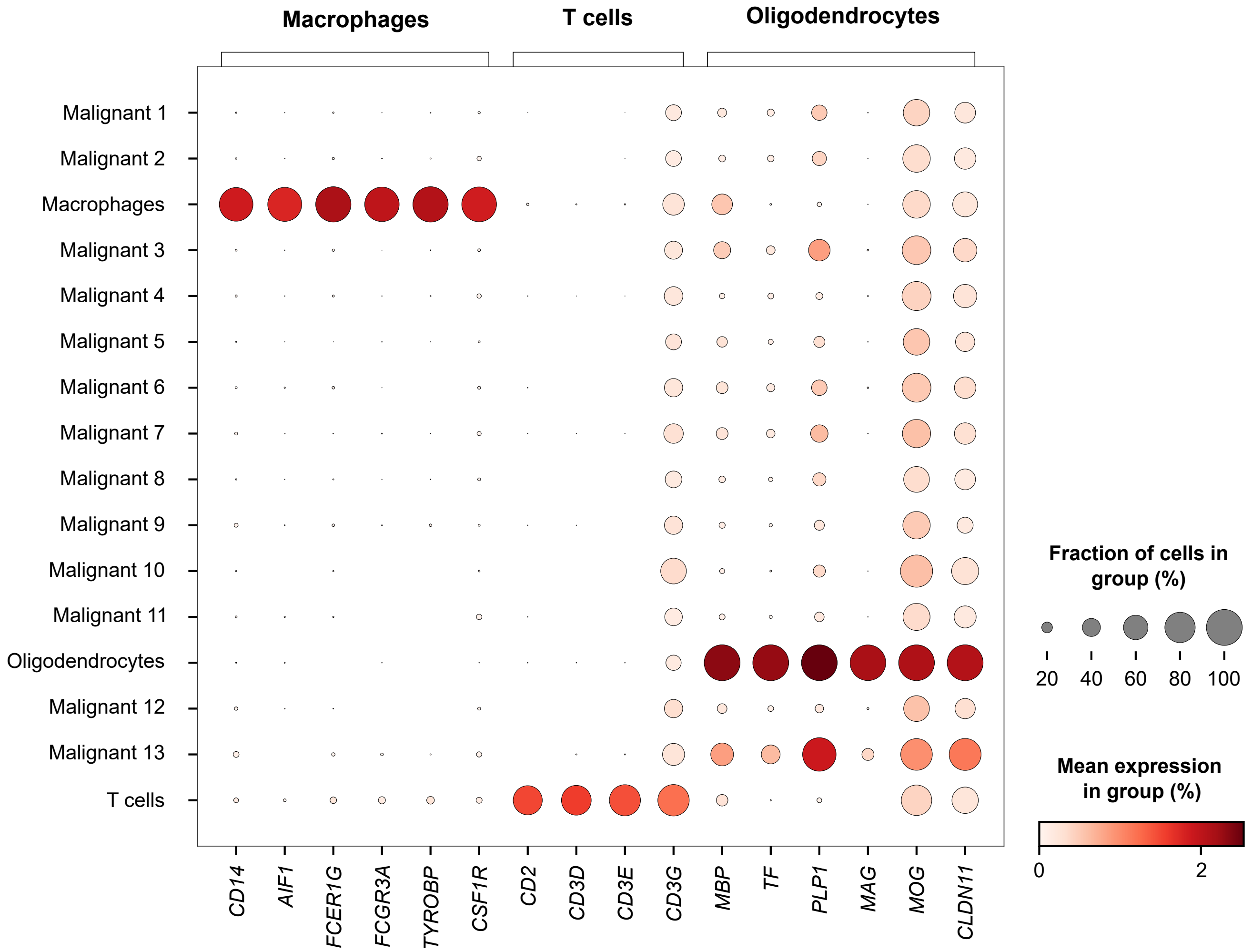

Macrophages
T cells
Oligodendrocytes
Malignant 1
Malignant 2
Macrophages
Malignant 3
Malignant 4
Malignant 5
Malignant 6
Malignant 7
Malignant 8
Malignant 9
Malignant 10
Malignant 11
Oligodendrocytes
Malignant 12
Malignant 13
T cells
CD14
AIF1
FCER1G
FCGR3A
TYROBP
CSF1R
CD2
CD3D
CD3E
CD3G
MBP
TF
PLP1
MAG
MOG
CLDN11
Fraction of cells in group (%)
20
40
60
80
100
Mean expression in group (%)
0
2

B

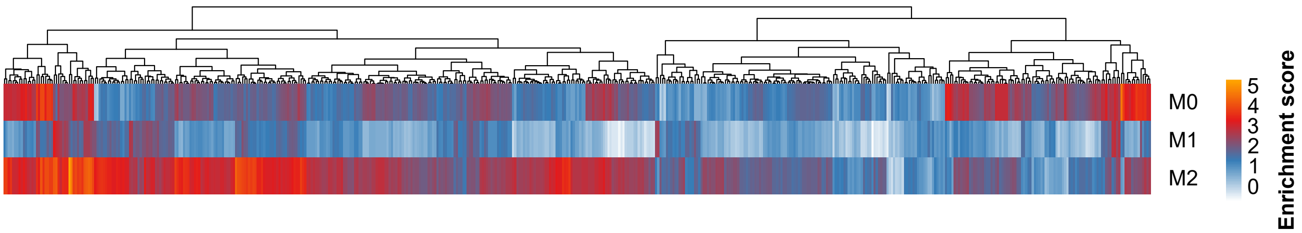

M0
M1
M2
5
4
3
2
1
0
Enrichment score

Figure S4. Expression of marker genes used to characterize the non-malignant cell populations, related to Figure 6.

(A) Bubble plot heatmap showing the mean expression of the select marker genes [7] used to characterize the non-malignant Louvain cell clusters of the scRNA-seq dataset (GSE131928). The red color intensity is directly proportional to the expression level of a marker gene in a specific cell population. The size of the circle denotes the fraction of cells expressing the marker gene.

(B) Heatmap showing the BPA scores of the macrophage signatures of the subsets M0, M1 and M2 in the scRNA-seq dataset (GSE131928).

Abbreviation: BPA: biological process activity; scRNA-seq: single cell RNA-sequencing.

Table S1. Transition parameters for Trp, related to Material and Methods.

Transition parameters used to measure Trp. The ion masses of parent and products after fragmentation are given, together with their respective collision energy (CE) values and the S-lens radio frequency levels. For each metabolite the six TMT® reporter ions and a metabolite specific fragment were monitored.

Analyte	Parent	Product	CE	S-lens
TMT® 126	434.26	126.13	29	172
TMT® 127	434.26	127.13	29	172
TMT® 128	434.26	128.13	29	172
TMT® 129	434.26	129.14	29	172
TMT® 130	434.26	130.14	29	172
TMT® 131	434.26	131.14	29	172
Fragment 188	434.26	188.07	28	172

Abbreviations: CE: collision energy; TMT®: tandem mass tag; Trp: tryptophan.

Table S2. Transition parameters for Kyn, related to Material and Methods.

Transition parameters used to measure Kyn. The ion masses of parent and products after fragmentation are given, together with their respective collision energy (CE) values and the S-lens radio frequency levels. For each metabolite the six TMT® reporter ions and a metabolite specific fragment were monitored.

Analyte	Parent	Product	CE	S-lens
TMT® 126	438.25	126.13	35	173
TMT® 127	438.25	127.13	35	173
TMT® 128	438.25	128.13	35	173
TMT® 129	438.25	129.14	35	173
TMT® 130	438.25	130.14	35	173
TMT® 131	438.25	131.14	35	173

Fragment 146	438.25	146.06	28	173
Abbreviations: CE: collision energy; Kyn: kynurenine; TMT®: tandem mass tag.				

Table S3. Transition parameters for FK, related to Material and Methods.

Transition parameters used to measure FK. The ion masses of parent and products after fragmentation are given, together with their respective collision energy (CE) values and the S-lens radio frequency levels. For each metabolite the six TMT® reporter ions and a metabolite specific fragment were monitored.

Analyte	Parent	Product	CE	S-lens
TMT® 126	466.25	126.13	30	150
TMT® 127	466.25	127.13	30	150
TMT® 128	466.25	128.13	30	150
TMT® 129	466.25	129.14	30	150
TMT® 130	466.25	130.14	30	150
TMT® 131	466.25	131.14	30	150
Fragment 174	466.25	174.05	30	150
Abbreviations: CE: collision energy; *N*-formylkynurenine; TMT®: tandem mass tag.				

Table S4. Transition parameters for OH-Trp, related to Material and Methods.

Transition parameters used to measure OH-Trp. The ion masses of parent and products after fragmentation are given, together with their respective collision energy (CE) values and the S-lens radio frequency levels. For each metabolite the six TMT® reporter ions and a metabolite specific fragment were monitored.

Analyte	Parent	Product	CE	S-lens
TMT® 126	450.25	126.13	30	150
TMT® 127	450.25	127.13	30	150
TMT® 128	450.25	128.13	30	150

TMT® 129	450.25	129.14	30	150
TMT® 130	450.25	130.14	30	150
TMT® 131	450.25	131.14	30	150
Fragment 175	450.25	175.08	30	150
Abbreviations: CE: collision energy; OH-Trp: hydroxy-tryptophan; TMT®: tandem mass tag.				

Table S5. Transition parameters for OH-Kyn, related to Material and Methods.

Transition parameters used to measure OH-Kyn. The ion masses of parent and products after fragmentation are given, together with their respective collision energy (CE) values and the S-lens radio frequency levels. For each metabolite the six TMT® reporter ions and a metabolite specific fragment were monitored.

Analyte	Parent	Product	CE	S-lens
TMT® 126	342.21	126.13	35	133
TMT® 127	342.21	127.13	35	133
TMT® 128	342.21	128.13	35	133
TMT® 129	342.21	129.14	35	133
TMT® 130	342.21	130.14	35	133
TMT® 131	342.21	131.14	35	133
Fragment 190	342.21	190.05	25	133
Abbreviations: CE: collision energy; OH-Kyn: hydroxy-kynurenine; TMT®: tandem mass tag.				

Table S6. Transition parameters for AA, related to Material and Methods.

Transition parameters used to measure AA. The ion masses of parent and products after fragmentation are given, together with their respective collision energy (CE) values and the S-lens radio frequency levels. For each metabolite the six TMT® reporter ions and a metabolite specific fragment were monitored.

Analyte	Parent	Product	CE	S-lens

TMT® 126	367.22	126.13	26	138
TMT® 127	367.22	127.13	26	138
TMT® 128	367.22	128.13	26	138
TMT® 129	367.22	129.14	26	138
TMT® 130	367.22	130.14	26	138
TMT® 131	367.22	131.14	26	138
Fragment 120	367.22	120.04	32	138

Abbreviations: AA: anthranilic acid; CE: collision energy; TMT®: tandem mass tag.

Table S7. Transition parameters for OH-AA, related to Material and Methods.

Transition parameters used to measure OH-AA. The ion masses of parent and products after fragmentation are given, together with their respective collision energy (CE) values and the S-lens radio frequency levels. For each metabolite the six TMT® reporter ions and a metabolite specific fragment were monitored.

Analyte	Parent	Product	CE	S-lens
TMT® 126	383.21	126.13	26	142
TMT® 127	383.21	127.13	26	142
TMT® 128	383.21	128.13	26	142
TMT® 129	383.21	129.14	26	142
TMT® 130	383.21	130.14	26	142
TMT® 131	383.21	131.14	26	142
Fragment 136	383.21	136.04	27	142

Abbreviations: CE: collision energy; OH-AA: hydroxy-anthranilic acid; TMT®: tandem mass tag.

Table S8. CV values of the MS/MS measurements of Trp and its metabolites, related to Material and Methods.

Representation of the mean coefficient of variation (CV) values and their SD (SD (CV)) for the different metabolites in the MS/MS measurements.

Metabolite	CV mean [%]	SD (CV) [%]
Trp	2.7	1.6
OH-Trp	2.4	2.0
FK	1.8	1.6
Kyn	2.4	1.6
AA	2.9	3.5
OH-Kyn	3.8	3.6
OH-AA	5.8	4.4

Abbreviations: AA: anthranilic acid; CV: coefficient of variation; FK: *N*-formylkynurenine; Kyn: kynurenine; MS/MS: tandem mass spectrometry; OH-AA: hydroxy-anthranilic acid; OH-Kyn: hydroxy-kynurenine; OH-Trp: hydroxy-tryptophan; SD: standard deviation; Trp: tryptophan.

Table S9. Overview of the characteristics of the study cohort for serum metabolite measurements, related to Figure 3.

	Patients	Controls
n	43	43
**Median age in years**	54.11	54.34
**Sex**	F: 19<br>M: 24	F: 19<br>M: 24
**IDH status**	WT: 33<br>Unknown: 10	
**Prior bevacizumab treatment**	yes: 29<br>no: 14	

Abbreviations: F: female; IDH: isocitrate dehydrogenase; M: male; WT: wild type.

Table S10. Detailed patient characteristics of the study cohort for serum metabolite measurements, related to Figure 3.

Patient number	MGMT promotor status	EGFR amplifi-cation	Evidence for PTEN loss	Timespan x between last surgery and blood draw [years]	Previous RT and TMZ treat-ment	Previous second line therapy with nitrosourea	Previ-ous ICB	Bevacizumab treatment before blood draw
1	n.a.	n.a.	n.a.	1.0 < x ≤ 1.5	yes	no	no	yes
2	methylated	n.a.	n.a.	1.5 < x ≤ 2.5	yes	yes	yes	yes
3	n.a.	n.a.	n.a.	2.5 < x ≤ 6.5	yes	no	yes	no
4	n.a.	n.a.	n.a.	1.5 < x ≤ 2.5	yes	yes	no	yes
5	unmethylated	not amplified	yes	0.5 < x ≤ 1.0	yes	no	no	yes
6	n.a.	n.a.	n.a.	1.5 < x ≤ 2.5	yes	yes	no	yes
7	n.a.	n.a.	n.a.	2.5 < x ≤ 6.5	yes	yes	no	yes
8	unmethylated	amplified	no	0.5 < x ≤ 1.0	yes	yes	yes	yes
9	n.a.	n.a.	no	1.0 < x ≤ 1.5	yes	no	no	yes
10	methylated	n.a.	n.a.	2.5 < x ≤ 6.5	yes	no	no	yes
11	methylated	not amplified	n.a.	0.5 < x ≤ 1.0	yes	no	no	yes
12	unmethylated	not amplified	yes	1.0 < x ≤ 1.5	yes	yes	no	yes
13	n.a.	n.a.	n.a.	1.0 < x ≤ 1.5	yes	no	no	yes
14	unmethylated	n.a.	n.a.	2.5 < x ≤ 6.5	yes	no	no	yes
15	n.a.	n.a.	n.a.	1.5 < x ≤ 2.5	yes	yes	no	yes
16	unmethylated	not amplified	yes	0.5 < x ≤ 1.0	yes	yes	no	yes
17	unmethylated	amplified	yes	1.0 < x ≤ 1.5	yes	yes	no	yes
18	unmethylated	not amplified	n.a.	0.5 < x ≤ 1.0	yes	no	no	yes
19	methylated	amplified	no	1.0 < x ≤ 1.5	yes	yes	no	no
20	unmethylated	not amplified	n.a.	1.0 < x ≤ 1.5	yes	no	no	no

21	unmethylated	n.a.	n.a.	0.5 < x ≤ 1.0	yes	yes	no	no
22	methylated	amplified	n.a.	1.0 < x ≤ 1.5	yes	yes	no	yes
23	n.a.	n.a.	n.a.	0 < x ≤ 0.5	yes	no	no	yes
24	n.a.	n.a.	n.a.	1.0 < x ≤ 1.5	yes	yes	no	yes
25	methylated	n.a.	no	0 < x ≤ 0.5	yes	yes	no	yes
26	methylated	not amplified	yes	1.5 < x ≤ 2.5	yes	no	no	yes
27	unmethylated	n.a.	yes	1.0 < x ≤ 1.5	yes	no	no	yes
28	unmethylated	n.a.	n.a.	1.0 < x ≤ 1.5	only RT	no	no	no
29	n.a.	n.a.	n.a.	2.5 < x ≤ 6.5	yes	yes	no	yes
30	methylated	n.a.	n.a.	0 < x ≤ 0.5	yes	no	no	no
31	n.a.	n.a.	n.a.	1.5 < x ≤ 2.5	yes	no	no	no
32	methylated	n.a.	n.a.	2.5 < x ≤ 6.5	yes	no	no	yes
33	methylated	not amplified	yes	0 < x ≤ 0.5	yes	no	no	no
34	methylated	n.a.	n.a.	0 < x ≤ 0.5	yes	yes	no	no
35	unmethylated	amplified	yes	0 < x ≤ 0.5	yes	no	no	no
36	methylated	not amplified	n.a.	0.5 < x ≤ 1.0	yes	no	no	no
37	n.a.	n.a.	no	1.0 < x ≤ 1.5	yes	yes	no	yes
38	methylated	amplified	yes	0 < x ≤ 0.5	yes	yes	no	yes
39	methylated	not amplified	yes	0 < x ≤ 0.5	yes	no	no	no
40	methylated	n.a.	n.a.	0 < x ≤ 0.5	yes	no	no	no
41	unmethylated	not amplified	yes	0 < x ≤ 0.5	yes	yes	no	yes
42	n.a.	n.a.	n.a.	0.5 < x ≤ 1.0	yes	no	no	no
43	unmethylated	not amplified	no	0 < x ≤ 0.5	yes	no	no	yes

Abbreviations: EGFR: epidermal growth factor receptor; ICB: immune checkpoint blockade; MGMT: O^6-methylguanine-DNA methyltransferase; n.a.: not applicable, clinical data not available; PTEN: phosphatase and tensin homolog; RT: radiotherapy; TMZ: temozolomide.

Table S11. Prior bevacizumab treatment does not influence Trp metabolite levels in serum, related to Figure 3.

Representation of the mean metabolite abundance in the sera of glioblastoma patient groups having received prior therapy with bevacizumab or not relative to reference sample depicted as log_2Ratio. Group comparisons were performed using a two-tailed unpaired Student's t test, p values are given as numbers.

Metabolite	Bevacizumab treatment prior to blood draw (*n* = 29)	No bevacizumab treatment prior to blood draw (*n* = 14)	P value
Trp	-0.23929	-0.171806	0.541873
OH-Trp	-0.20438	-0.147861	0.590434
FK	-0.18088	-0.128576	0.623908
Kyn	-0.30523	-0.296833	0.943081
AA	-0.19042	-0.203277	0.861635
OH-Kyn	0.54967	0.431191	0.376339
OH-AA	-0.21835	-0.074910	0.365114
Abbreviations: AA: anthranilic acid; FK: *N*-formylkynurenine; Kyn: kynurenine; OH-AA: hydroxy-anthranilic acid; OH-Kyn: hydroxy-kynurenine; OH-Trp: hydroxy-tryptophan; Trp: tryptophan.			

Table S12. MGMT promotor methylation status does not influence Trp metabolite levels in serum, related to Figure 3.

Representation of the mean metabolite abundance in the sera of glioblastoma patient groups with a methylated or unmethylated MGMT promotor relative to reference sample depicted as log_2Ratio. Group comparisons were performed using a two-tailed unpaired Student's t test, p values are given as numbers.

Metabolite	methylated MGMT promotor (*n* = 15)	unmethylated MGMT promotor (*n* = 14)	P value
Trp	-0.307804	-0.23550	0.606817
OH-Trp	-0.290598	-0.17417	0.379436
FK	-0.244386	-0.19412	0.710315
Kyn	-0.318028	-0.36445	0.737927

AA	-0.269437	-0.19970	0.408155
OH-Kyn	0.552551	0.42303	0.412657
OH-AA	-0.165172	-0.06865	0.568299
Abbreviations: AA: anthranilic acid; FK: *N*-formylkynurenine; Kyn: kynurenine; MGMT: O^6-methylguanine-DNA methyltransferase; OH-AA: hydroxy-anthranilic acid; OH-Kyn: hydroxy-kynurenine; OH-Trp: hydroxy-tryptophan; Trp: tryptophan.			

Table S13. EGFR amplification status does not influence Trp metabolite levels in serum, related to Figure 3.

Representation of the mean metabolite abundance in the sera of glioblastoma patient groups with EGFR amplification or not relative to reference sample depicted as log_2Ratio. Group comparisons were performed using a two-tailed unpaired Student's t test, p values are given as numbers.

**Metabolite**	**EGFR amplification (*n* = 6)**	**No EGFR amplification (*n* = 12)**	**P value**
Trp	-0.284102	-0.36048	0.682655
OH-Trp	-0.190309	-0.33811	0.413630
FK	-0.226813	-0.31161	0.635678
Kyn	-0.333124	-0.42910	0.589516
AA	-0.264457	-0.30276	0.730573
OH-Kyn	0.526196	0.34979	0.392807
OH-AA	-0.264156	-0.17875	0.709499
Abbreviations: AA: anthranilic acid; EGFR: epidermal growth factor receptor; FK: *N*-formylkynurenine; Kyn: kynurenine; OH-AA: hydroxy-anthranilic acid; OH-Kyn: hydroxy-kynurenine; OH-Trp: hydroxy-tryptophan; Trp: tryptophan.			

Table S14. PTEN loss does not influence Trp metabolite levels in serum, related to Figure 3.

Representation of the mean metabolite abundance in the sera of glioblastoma patient groups with evidence for PTEN loss or not relative to reference sample depicted as log_2Ratio. Group comparisons were performed using a two-tailed unpaired Student's t test, p values are given as numbers.

Metabolite	Evidence for PTEN loss (n = 11)	No evidence for PTEN loss (n = 6)	P value
Trp	-0.395536	-0.24675	0.429786
OH-Trp	-0.333291	-0.16009	0.360572
FK	-0.350850	-0.15310	0.272037
Kyn	-0.464462	-0.19459	0.134742
AA	-0.300172	-0.21243	0.447791
OH-Kyn	0.440620	0.64832	0.248894
OH-AA	-0.117671	-0.51601	0.064070

Abbreviations: AA: anthranilic acid; FK: *N*-formylkynurenine; Kyn: kynurenine; OH-AA: hydroxy-anthranilic acid; OH-Kyn: hydroxy-kynurenine; OH-Trp: hydroxy-tryptophan; PTEN: phosphatase and tensin homolog; Trp: tryptophan.

Table S15. Reduction of metabolite levels in glioblastoma patient sera, related to Figure 4.

Representation of the $\log_2$ fold change (FC) values of metabolite level reduction in patient versus age- and sex-matched control sera as depicted in Figure 4B and the corresponding reduction of metabolite levels given in %.

**Metabolite**	**$\mathbf{Log_2}$ (FC patients vs controls)**	**FC (patients vs controls)**	**Metabolite level reduction in patients vs controls (%)**	**P value**
Trp	-0.300	0.812	18.8	0.000261358
OH-Trp	-0.268	0.830	17.0	0.000460516
FK	-0.273	0.827	17.3	0.000558024
Kyn	-0.219	0.859	14.1	0.010150130
AA	-0.164	0.892	10.8	0.007304935
OH-Kyn	-0.042	0.971	2.9	0.686404933
OH-AA	-0.007	0.995	0.5	0.952159761

Abbreviations: AA: anthranilic acid; FC: fold change; FK: *N*-formylkynurenine; Kyn: kynurenine; OH-AA: hydroxy-anthranilic acid; OH-Kyn: hydroxy-kynurenine; OH-Trp: hydroxy-tryptophan; Trp: tryptophan; vs: versus.

Table S16. Characteristics of tumor tissue samples analyzed with MALDI MSI, related to Figure 5 and Figure S3.

Patient number	Primary glioblastoma	IDH status	MGMT promotor status	EGFR amplification	Evidence for PTEN loss
44	primary	WT	methylated	amplified	n.a.
45	primary	WT	methylated	not amplified	n.a.
46	primary	WT	unmethylated	not amplified	n.a.
47	primary	WT	unmethylated	not amplified	no
48	primary	WT	unmethylated	amplified	no
49	primary	WT	methylated	not amplified	n.a.
50	primary	WT	unmethylated	not amplified	no
51	primary	WT	unmethylated	not amplified	no
52	primary	WT	methylated	amplified	n.a.
53	primary	WT	unmethylated	amplified	yes
54	primary	WT	unmethylated	n.a.	yes
55	n.a.	WT	unmethylated	not amplified	no
56	primary	WT	methylated	not amplified	no
57	n.a.	n.a.	n.a.	n.a.	n.a.
58	primary	WT	methylated	not amplified	no
59	primary	n.a.	n.a.	not amplified	n.a.
60	primary	WT	methylated	not amplified	no

Abbreviations: EGFR: epidermal growth factor receptor; IDH: isocitrate dehydrogenase; MALDI MSI: MALDI mass spectrometry imaging; MGMT: O^6-methylguanine-DNA methyltransferase; n.a.: not applicable, clinical data not available; PTEN: phosphatase and tensin homolog; WT: wild type.

Table S17. AHR activity associates with worse overall survival in glioblastoma patients, related to Figure 6.

Univariate and multivariate cox regression analysis of the effect of AHR activity and age at diagnosis on overall survival in glioblastoma patients in the TCGA database.

**Univariate analysis**				
	**Coef**	**Se.coef.**	**Z**	**P value**
Age at diagnosis	9.60E-05	2.44E-05	3.930614673	8.47E-05
AHR activity	0.190344934	0.092285956	2.06255581	0.03915485
**Multivariate analysis**				
	**Coef**	**Se.coef.**	**Z**	**P value**
Age at diagnosis	9.75E-05	2.49E-05	3.912168313	9.15E-05
AHR activity	0.187806232	0.092976393	2.019934585	0.043390173
Abbreviations: AHR: aryl hydrocarbon receptor; Coef: coefficient; Se.coef.: Standard error of the coefficient; Z: Wald test z score, which is the coefficient divided by its standard error.				
